# O-Methylation in Carbohydrates: An NMR and MD Simulation Study with Application to Methylcellulose

**DOI:** 10.1021/acs.jpcb.1c07293

**Published:** 2021-10-27

**Authors:** Alessandro Ruda, Göran Widmalm, Jakob Wohlert

**Affiliations:** † Department of Organic Chemistry, Arrhenius Laboratory, Stockholm University, SE-106 91 Stockholm, Sweden; ‡ Department of Fiber and Polymer Technology, School of Chemistry, Biotechnology and Health, KTH Royal Institute of Technology, SE-100 44 Stockholm, Sweden; § Wallenberg Wood Science Center, KTH Royal Institute of Technology, SE-100 44 Stockholm, Sweden

## Abstract

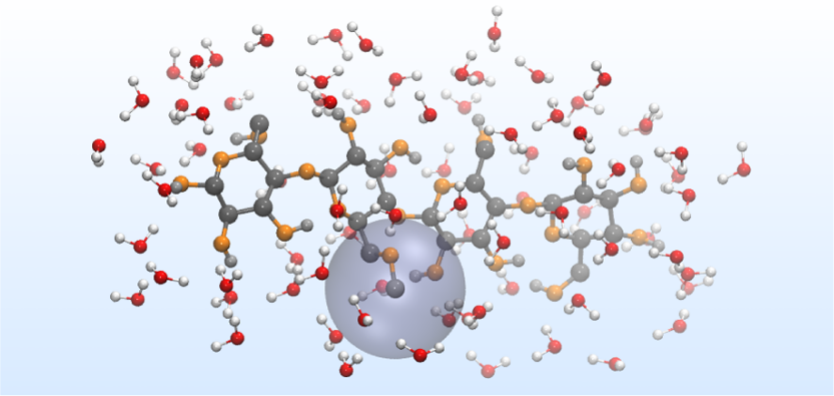

Methylated carbohydrates are important from both biological and technical perspectives. Specifically, methylcellulose is an interesting cellulose derivative that has applications in foods, materials, cosmetics, and many other fields. While the molecular dynamics simulation technique has the potential for both advancing the fundamental understanding of this polymer and aiding in the development of specific applications, a general drawback is the lack of experimentally validated interaction potentials for the methylated moieties. In the present study, simulations using the GROMOS 56 carbohydrate force field are compared to NMR spin–spin coupling constants related to the conformation of the exocyclic torsion angle ω in d-glucopyranose and derivatives containing a 6-O-methyl substituent and a ^13^C-isotopologue thereof. A ^3^*J*_CC_ Karplus-type relationship is proposed for the C5–C6–O6–C_Me_ torsion angle. Moreover, solvation free energies are compared to experimental data for small model compounds. Alkylation in the form of 6-O-methylation affects exocyclic torsion only marginally. Computed solvation free energies between nonmethylated and methylated molecules were internally consistent, which validates the application of these interaction potentials for more specialized purposes.

## Introduction

Methylation in carbohydrates is found in many places in nature such as bacteria, fungi, worms, mollusks, algae, and plants, but it is still rare compared to other modifications.^1^ Methylated oligosaccharides are only found in bacteria and plants, where for instance certain plant hemicelluloses (xylans) are rich in 4-O-methylated glucuronic acids,^2^ and 6-O-methyl substitutions are present in glucosyl residues from mycobacterial polysaccharides^3−6^ and in a polysaccharide from the leaves of the plant *Catharanthus rosea*.^7^

**Figure 1:**
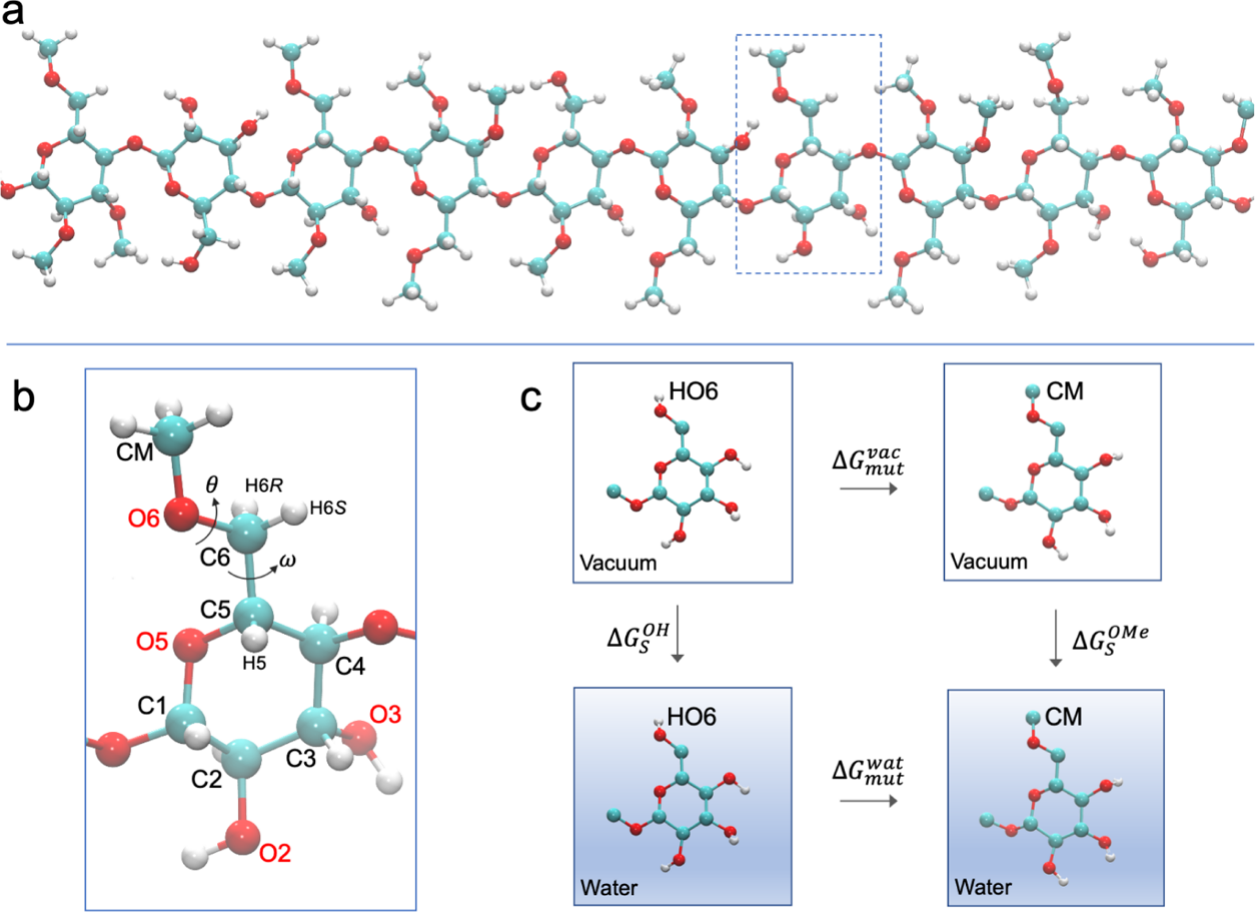
(a) Illustration of a MC chain in an all-atom representation which is randomly 2-, 3-, and 6-O-methylated. (b) Close-up of a 6-O-methylated glucopyranose unit with the notations for atoms and torsion angles used in this article. (c) Thermodynamic cycle with the arrows defining the sign of the corresponding free energy change. Vertical paths represent solvation free energies and horizontal paths represent the mutation of β-d-Glc*p*-OMe into β-d-Glc*p*6Me-OMe, respectively. Note that the monosaccharides are drawn in the GROMOS united atoms representation (i.e. without explicit aliphatic hydrogen atoms), which is used in the simulations.

Much is still not known about the specific function of methylation in living organisms, although it seems to be connected to molecular recognition.^1^ In addition, it also provides variation in chemical properties by rendering the carbohydrate more hydrophobic, and also by modulating the conformational space.^8^ This is exploited in methylcellulose (MC) which is synthesized from cellulose by O-methylation in positions 2, 3, and/or 6 of the glucose units (Figure 1a). Being a cellulose derivative, MC belongs to an important class of biopolymers that are renewable, nontoxic, easy to synthesize, cheap, industrially viable, and in most cases water-soluble (in contrast to cellulose) with an interesting phase behavior. Thus, MC is used in foods, cosmetics, pharmaceutics, construction materials, adhesives, drilling fluids, and so forth.^9^ As a consequence of the synthesis route, commercial MCs are characterized by a heterogeneous distribution of methyl groups along the polymer chain, while homogeneous methylation can also be achieved through dissolution prior to the methylation reaction.

Despite often being described as a hydrophilic polymer, cellulose is completely insoluble in water, where it forms crystalline aggregates that are characterized by an extensive hydrogen bond network. However, as these internal hydrogen bonds are of comparable energy as potential hydrogen bonds with water molecules, hydrogen bonding is not the thermodynamic driving force for cellulose aggregation. Instead, the free energy is dominated by the large penalty of hydrating the pseudo-flat apolar surfaces of the glucose rings, a.k.a. the hydrophobic effect.^10,11^ O-methylation disrupts the hydrogen bond network that allows for close packing of the glucan chains and tips the energy balance in favor of dissolution. However, if the methylation is driven too far, hydrophobic effects again start to dominate, and the MC polymer becomes insoluble. Depending on the synthesis route, MCs with a degree of substitution (DS) between 0.9 and 2.5 are water-soluble at room temperature and below.^9^ However, if the temperature increases above a lower critical solution temperature of 303 K, MC chains start to associate, and above approximately 323 K, phase separation starts to occur, eventually leading to gel formation.^12,13^ Several mechanisms for the thermal behavior of MC have been proposed, so far without a clear consensus having been reached. Most of them invoke the heterogeneous distribution of methyl groups as a key factor, which leads to the formation of network junctions between highly methylated sections as a result of hydrophobic association at elevated temperatures,^12^ and indeed, homogenously methylated cellulose does not exhibit the sol–gel transition.^14^

Solution state NMR is an invaluable tool for studying molecular structure of glycans,^15,16^ for instance, through the assignment of population-averaged rotational states from spin–spin couplings using Karplus-type relationships.^17^ While numerous Karplus equations for monosaccharides and their derivatives have been published, an appropriate equation for the θ torsion angle (Figure 1b) for 6-O-methylated compounds is missing. In addition, molecular dynamics (MD) simulation has evolved into an important tool for studying physical properties of biomolecules, especially in combination with experimental methods, such as NMR spectroscopy. Several of the most-used force fields for MD simulation of carbohydrates, such as CHARMM,^18^ GLYCAM,^19^ and GROMOS,^20^ provide patches or general rules for the inclusion of common derivatizations, including methylation. However, such modifications were not specifically targeted in the force field optimization, and have thus not, from what it appears, been as extensively validated as the unsubstituted sugars. Nevertheless, MD has been used to study methylated carbohydrates and carbohydrate polymers in solution, although reports on simulations of MC are scarce. Most notably, Larson et al. have used both atomistic and coarse-grained MD to study the phase behavior of MC,^21−23^ and Yu et al. studied the effect of O-methylation in cellooligomers^24^ on both structure and solvation properties, while Delbianco and co-workers^25^ studied conformational properties systematically with respect to various modifications, including O-methylation.

In this work, the solution structure of 6-O-methylated glucopyranosides is investigated by NMR and MD simulations. A new ^3^*J*_CC_ Karplus-type relation for the exo-cyclic θ torsion is parameterized. This equation is used to validate simulations using the GROMOS carbohydrate force field.^20^ Simulations are further extended to include selectively methylated cellooligomers with the purpose of studying the effect on the free energy of hydration. These results are compared to experimental data for methylated model compounds in solution.

## Methods

### Saccharides

**Figure 2:**
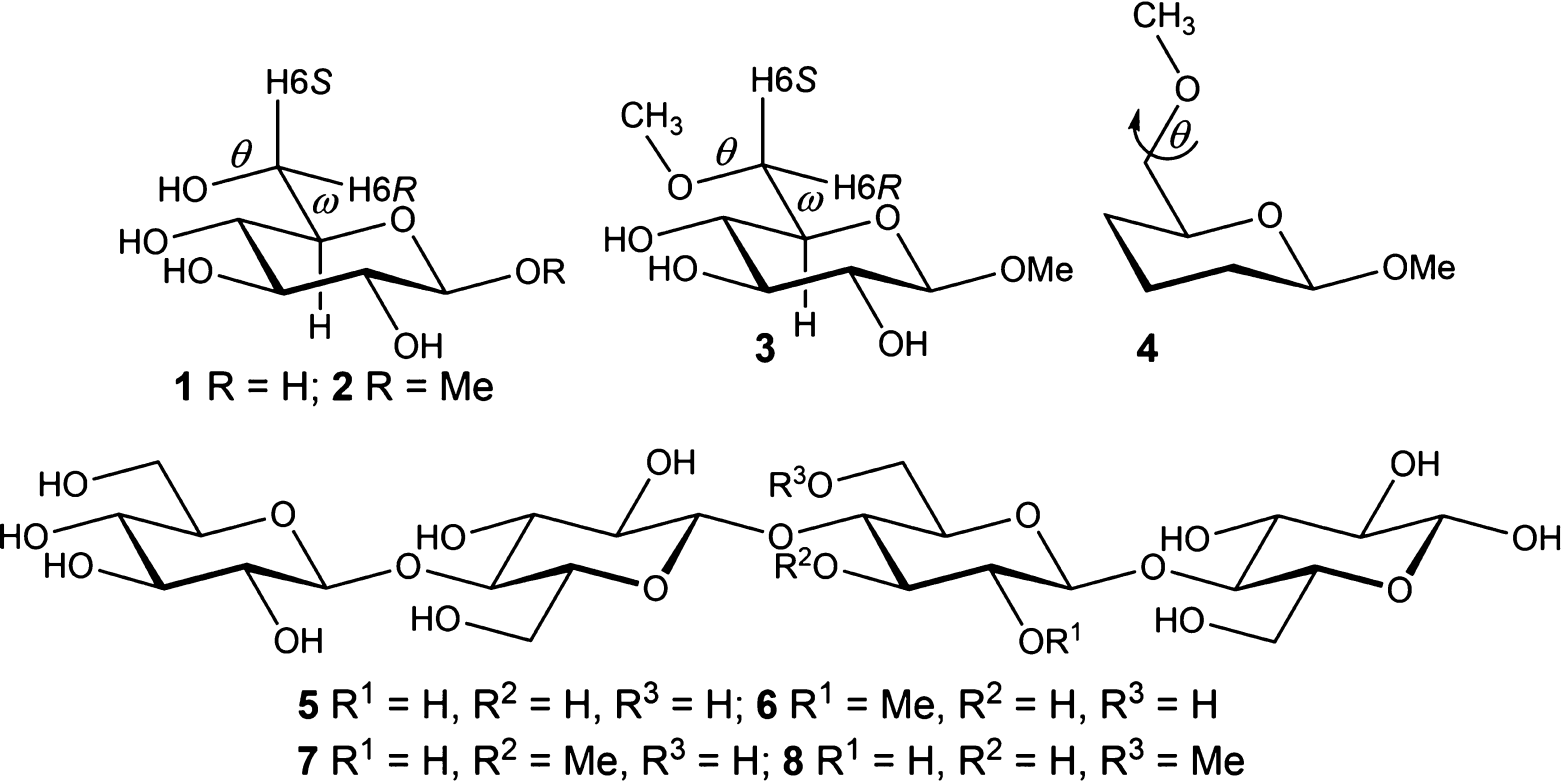
Schematic representation of model substances: (top) β-d-Glc*p* (**1**) or β-d-Glc*p*-OMe (**2**), β-d-Glc*p*6Me-OMe (**3**), (2*R*,6*S*)-2-methoxy-6-(methoxymethyl)tetrahydro-2*H*-pyran (**4**), (bottom) β-cellotetraose (**5**), and β-cellotetraose O-methylated in the C2, C3, or C6 position (**6–8**). The exo-cyclic torsion angles ω and θ are indicated. Hydroxymethyl protons H6_pro-*R*_ and H6_pro-*S*_ are denoted by H6*R* and H6*S*, respectively.

The carbohydrate model compounds used were the three monosaccharides β-d-Glc*p*, β-d-Glc*p*-OMe and β-d-Glc*p*6Me-OMe, and cellotetraose, which was selectively 2-, 3-, or 6-O-methylated (Figure 2). The synthesis of the compounds used in NMR experiments is described in Supporting Information.

### NMR Spectroscopy

NMR experiments for conformational analysis utilized monosaccharides dissolved in D_2_O (99.96%) at a concentration of 30–40 mM. The NMR spectra were recorded on Bruker spectrometers, viz., an AVANCE III 700 MHz equipped with a 5 mm TCI Z-Gradient Cryoprobe (^1^H/^13^C/^15^N) or an AVANCE III 600 MHz equipped with a 5 mm TXI inverse Z-Gradient ^1^H/^13^C/^31^P probe. The chemical shifts were referenced to internal sodium 3-trimethylsilyl-(2,2,3,3-^2^H_4_)-propanoate (TSP) in D_2_O (δ_H_ 0.0) and external 10% 1,4-dioxane in D_2_O (δ_C_ 67.4). ^1^H and ^13^C NMR experiments were recorded at 298 K, unless otherwise stated, with a digital resolution of 0.2 and 0.5 Hz/point, respectively, and zero-filled prior to Fourier transformation of the FIDs. ^*n*^*J*_HH_ and ^3^*J*_CH_ coupling constants were extracted by lineshape analysis using the NMR spin-simulation software PERCH;^26^ the experimental error for ^*n*^*J*_HH_ is estimated to be ≤0.2 Hz. The homonuclear broadband-decoupled pure shift ^1^H NMR spectrum^27^ of β-d-Glc*p*6[^13^C]Me-OMe (**3*c***) was recorded at 600 MHz using the SAPPHIRE method, in which sideband artefacts were suppressed by averaging using periodic phase incrementation of residual *J* evolution.^28^ A spectral width of 6 kHz was sampled with 16k data points in the direct dimension in conjunction with 16 chunks with a spectral window of 39.279 Hz in the indirect dimension using eight phase incrementations per chunk for sideband suppression. The final spectrum originating from concatenation of the 16 chunks was zero-filled to 32k data points prior to Fourier transformation. A ^1^H-detected one-dimensional long-range (1DLR) experiment^29^ with pulsed-field-gradients^30^ was used to determine the ^3^*J*_C4,H6_ coupling constants in compound **3** employing 32k data points per FID, an acquisition time of 2 s and a digital resolution of 0.5 Hz per data point. A nominal long-range coupling constant was set to a value of 6 Hz and a Gaus1_90.1000 soft pulse was used for ^13^C resonance excitation, calibrated corresponding to a 90° high power pulse duration of 14.8 μs. The duration of the soft pulse was chosen accordingly to the degree of selectivity needed. 1D ^1^H,^1^H-NOESY experiments^31^ with 10 different mixing times from 50 to 800 ms were used with an acquisition time of 4 s and a 120 ms Rsnob.1000 shaped pulse for selective excitation of the H4 resonance in **3*c***; suppression of zero-quantum interferences employed a 20 ms Chirp adiabatic pulse and a gradient strength at 5% of its maximum. NMR spectra were processed and analyzed using TopSpin 4.0.1 software (Bruker BioSpin). Subsequent to zero-filling prior to Fourier transformation of the FID extraction of the ^3^*J*_C4,H6*S*_ coupling constant in **3** was performed by the *J* doubling procedure in the frequency domain^32^ using an in-house script implemented in MATLAB R2017a (MathWorks), first with a delta function for in-phase multiplets of [−1 1 −1 1 1 −1 1 −1] spaced by a trial coupling constant J* corresponding to the in-phase vicinal and subsequently geminal coupling and then for the anti-phase doublet with a delta function of [1 1 1 1 −1 −1 −1 −1], in *J* increments of 0.1 Hz; the experimental error for ^*n*^*J*_CH_ is estimated to be ≤0.2 Hz.

### Karplus Relations

The Karplus relations, with resulting scalar coupling constants in Hz, used in this article are given below together with their references. The torsion angles are defined from the sequences (Figure 1b) O5–C5–C6–O6 (ω), C4–C5–C6–O6 (ω_C_), C4–C5–C6–H6*R* alternatively C4–C5–C6–H6*S* (ω_CH_), C5–C6–O6–H_O6_ alternatively C5–C6–O6–C_Me_ (θ), H6*R*–C6–O6–C_M_ alternatively H6*S*–C6–O6–C_Me_ (θ_H_).

Stenutz et al. gave the following equations:^33^
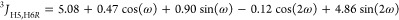
1
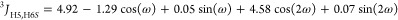
2

Thibaudeau et al. gave the following equation:^34^
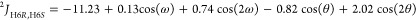
3

Meredith et al. gave the following equations:^35^
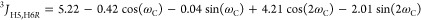
4
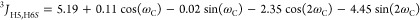
5
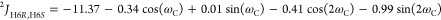
6

Tvaroška, Hrikovíni, and Petráková gave the following equation:^36^
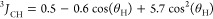
7

Thibaudeau et al. gave the following equations:^34^
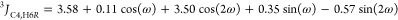
8
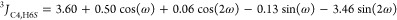
9

Tvaroška and Gajdoš gave the following equation:^37^
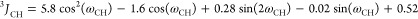
10

Watson et al. gave the following equations:^38^
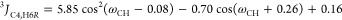
11
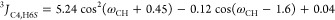
12where the phase-shifts in the latter two equations are given in radians (eq 12 has been corrected to a plus sign in the first term; personal communication with Dr A. H. Franz). Plots of all equations are shown in Supporting Information.

### Molecular Dynamics Simulations

The computational model for methylated carbohydrates was built on the GROMOS carbohydrate force field,^20^ together with the SPC/E^39^ potential for water. This force field was previously shown to perform better than both CHARMM35^18^ and GLYCAM06^19^ with respect to the hydroxymethyl conformation of cellooligomers in water; specifically it reproduces the experimentally determined weak dependence on temperature.^40^ Simulations of methanol and dimethyl ether (DME) used parameters from the ATB repository^41^ (IDs 15607 and 21337, respectively), which are based on the GROMOS 54A7 force field.^42^ All molecular dynamics simulations were run using GROMACS^43^ version 2016 (or later) using a basic time step of 2 fs. Nonbonded interactions were cut off at 1.2 nm, and the long-range electrostatics was included using PME.^44,45^ To improve sampling, *all* simulations were run using a replica-exchange protocol,^46^ with 12 replicas at different temperatures ranging from 298 to 364 K in 6 K increments, for which exchanges between neighboring replicas were attempted every 100 steps. Unless stated otherwise, properties calculated from the 298 K trajectory are reported. The temperatures were controlled by stochastic velocity rescaling^47^ and the pressure was maintained at 1 atm using the Parrinello–Rahman barostat,^48^ except for vacuum simulations which were run at constant volume where the temperature was controlled by the stochastic integration algorithm.^49^

### Calculation of Free Energies

The free energies between two states (A and B) can be calculated using *computational alchemy*(50) where the Hamiltonian is parameterized by a single parameter λ such that λ = 0 corresponds to state A, and λ = 1 corresponds to state B. The system can then be sampled at several intermediate values of λ using separate simulations, and finally connected using, for example, Bennet’s acceptance ratio^51^ to give the free energy profile between the states. Here, to calculate free energies of solvation (Δ*G*_s_), the states were chosen such that λ = 0 corresponded to a state where all solute–solvent interactions were intact, and λ = 1 to a state where they were completely decoupled. The free energy difference in this case (going from λ = 0 to λ = 1) is the free energy of removing the solute from the solvent, into its vapor phase (−Δ*G*_s_). These simulations used 20 λ-points, each simulated for 5 ns with replica exchange. Alternatively, the difference in solvation free energy, ΔΔ*G*_s_, between two different species (e.g., a nonmethylated and methylated carbohydrate) can be calculated using the same method, but where state A is the nonmethylated solute, and B is the methylated one (Figure 1c). Here, the transition between the end states involves the “mutation” of the hydroxyl proton into a CH_3_ group (which is a single interaction site in the GROMOS united atoms representation), with consequences on both nonbonded and bonded interaction terms. The difference between the free energies obtained in this way from simulations in both solvent and vacuum corresponds to ΔΔ*G*_s_between the original and the methylated solute, ΔΔ*G*_s_ = Δ*G*_*s*_^OMe^ – Δ*G*_s_^OH^ = Δ*G*_mut_^wat^ – Δ*G*_mut_^vac^, due to that the thermodynamic cycle must add up to zero (Figure 1c), and is thus a measure of the effect of methylation itself. For these simulations, 25 λ-points were used, each simulated for 2 ns, employing temperature replica exchange as described above. This protocol was recently used for simulations of acetylated cellooligomers and cellulose nanocrystals.^52^

## Results and Discussion

The monosaccharide glucose, methyl derivatives thereof and a pseudo-monosaccharide were chosen as models for NMR experiments and computational studies, viz., β-d-Glc*p* (**1**), β-d-Glc*p*-OMe (**2**), its 6-O-methylated derivative β-d-Glc*p*6Me-OMe (**3**), and the pyran derivative (**4**) (Figure 2). The 6-O-methylated glucosides were synthesized via a three-step procedure from the fully protected methyl 2,3-di-*O*-benzyl-4,6-*O*-benzylidine-β-d-glucopyranoside by regioselective ring-opening reduction of the 4,6-*O*-benzylidene group,^53^ alkylation with methyl iodide or [^13^C]methyl iodide, and removal of the benzyl ether groups^54^ to give β-d-Glc*p*6Me-OMe (**3**),^55^ and β-d-Glc*p*6[^13^C]Me-OMe (**3*c***), respectively (see Supporting Information). These 6-O-methylated monosaccharide derivatives of glucose facilitate a detailed analysis of conformational dynamics, related to entities of MC.

### NMR Experiments for Conformational Analysis of Monosaccharides

**Figure 3:**
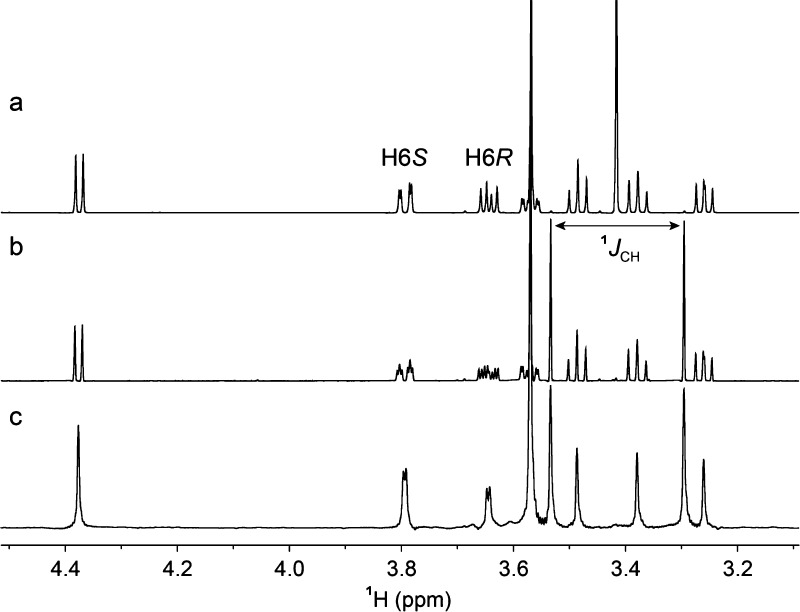
^1^H NMR spectra at 600 MHz of compounds in D_2_O at 298 K: (a) β-d-Glc*p*6Me-OMe (**3**), (b) β-d-Glc*p*6[^13^C]Me-OMe (**3*c***), and (c) pure shift ^1^H NMR spectrum of compound **3*c***.

**Figure 4:**
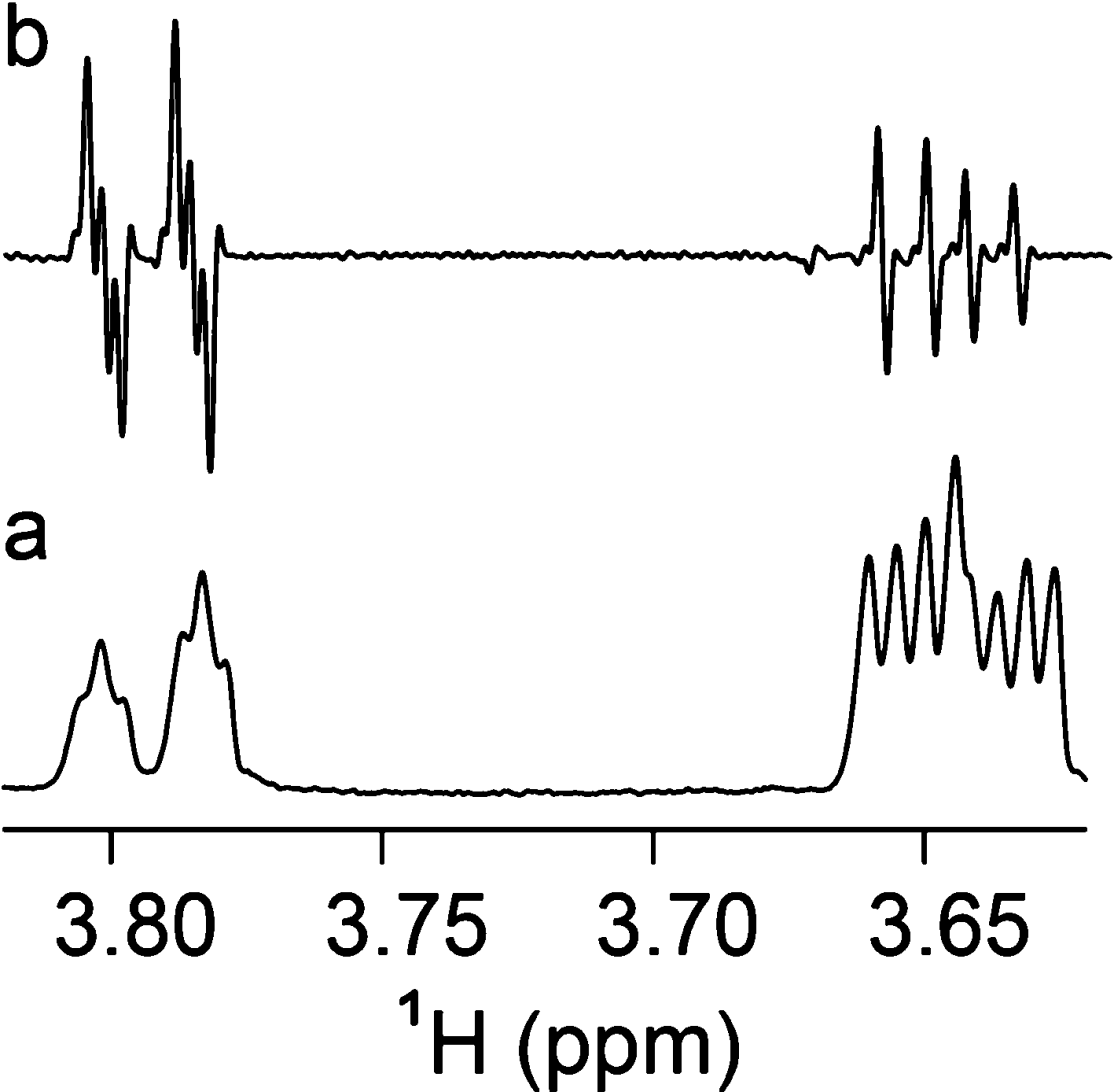
Selected ^1^H NMR spectral region of H6 protons of (a) a 1D ^1^H,^1^H-NOESY NMR spectrum at 700 MHz, with a mixing time of 800 ms, of β-d-Glc*p*6[^13^C]Me-OMe (**3*c***) in D_2_O at 298 K in which the H4 resonance was selectively excited and (b) a ^1^H,^13^C-heteronuclear one-dimensional long-range (1DLR) spectrum of the corresponding spectral region and experimental conditions for β-d-Glc*p*6Me-OMe (**3**) in which the C4 resonance was selectively excited.

The ^1^H NMR resonance assignments for H6*R* and H6*S* in β-d-Glc*p*-OMe are known based on selective deuteration methodology.^56^^3^*J*_H5,H6_, which may be used to determine the population distribution of the ω torsion angle, as well as ^2^*J*_H6*R*,H6*S*_ were determined by total lineshape analysis^26^ (Table 1), in full agreement with previously reported values.^56−58^ For β-d-Glc*p*6Me-OMe, the ^1^H NMR spectrum showed resolved resonances for the protons of the hydroxymethyl group (Figure 3a), and in β-d-Glc*p*6[^13^C]Me-OMe additional couplings were present; these scalar interactions originate from the ^13^C-labeled methyl group (Figure 3b), evident also in the pure shift ^1^H NMR spectrum (Figure 3c). To assign the resonances from H6*R* and H6*S* in β-d-Glc*p*6Me-OMe, a series of 1D ^1^H,^1^H-NOESY experiments were used with selective excitation of the H4 resonance, resulting for the resonances at 3.64 and 3.79 ppm in a relative ratio of 2.3:1 at the longest mixing time (Figure 4a). By considering that 1,3-diaxial interactions, corresponding to a *syn*-pentane relationship,^59,60^ between hydroxyl groups in sugars are energetically disfavored,^61^ the NOE results facilitate assignment of the resonance at 3.64 ppm to H6*R* and the one at 3.79 ppm to H6*S*. The ^1^H,^13^C-heteronuclear ^3^*J*_H6,Me_ in β-d-Glc*p*6[^13^C]Me-OMe were determined by the total lineshape fitting procedure (Table 1). The ^3^*J*_C4,H6_ coupling constants related to the ω_C_ torsion angle were obtained by a ^1^H-detected one-dimensional long-range (1DLR) experiment^29,30^ (Figure 4b) followed by extraction of the ^3^*J*_C4,H6*S*_ coupling constant by the *J* doubling procedure in the frequency domain^32^ resulting in a relatively large value of 2.5 Hz (Table 1). However, the ^3^*J*_C4,H6*R*_ coupling constant was smaller and from the anti-phase peak separation, its value was estimated^62^ to be <1.2 Hz (Table 1); these coupling constants are in excellent agreement with previously determined ^3^*J*_C4,H6_ values^58^ obtained by a different NMR experiment, and similar in magnitude to other glucopyranosides.^63,64^ The ^13^C,^13^C-homonuclear ^3^*J*_C5,Me_ coupling (Table 1) in β-d-Glc*p*6[^13^C]Me-OMe was readily determined from the doublet of the C5 resonance in the 1D ^13^C NMR spectrum.

**Table 1:** Experimental and Computed NMR Spin–Spin Coupling Constants (Hz) in β-d-Glc*p*-OMe (**2**) and β-d-Glc*p*6Me-OMe (**3**)

coupled nuclei	NMR	molecular dynamics		
Compound **2**				
H5,H6*R*	6.15	6.62^a^	6.28^c^	
H5,H6*S*	2.25	2.43^a^	2.94^c^	
H6*R*,H6*S*	–12.33	–11.23^b^	–11.65^c^	
C4,H6*R*	1.0^h^	2.12^b^	1.50^d^	1.65^e^
C4,H6*S*	2.4^h^	3.03^b^	3.45^d^	3.81^e^
Compound **3**				
H5,H6*R*	6.35	7.15^a^	6.71^c^	
H5,H6*S*	2.09	2.26^a^	2.71^c^	
H6*R*,H6*S*	–11.26	–9.92^b^	–11.66^c^	
Me,H6*R*	3.16^i^	2.64^f^		
Me,H6*S*	2.69^i^	2.20^f^		
Me,C5	3.4^i^	4.99^g^		
C4,H6*R*	<1.2^j^ (1.0)^h^	1.98^b^	1.39^d^	1.58^e^
C4,H6*S*	2.5^j^ (2.45)^h^	2.72^b^	3.15^d^	3.91^e^

a Karplus-type relationships used in the simulations:^3^*J*_HH_ from Stenutz et al.^33^

b Karplus-type relationships used in the simulations: ^2^*J*_HH_ and ^3^*J*_CH_ from Thibaudeau et al.^34^

c Karplus-type relationships used in the simulations: ^*n*^*J*_HH_ from Meredith et al.^35^

d Karplus-type relationships used in the simulations: ^3^*J*_CH_ from Tvaroška et al. (1995).^37^

e Karplus-type relationships used in the simulations: ^3^*J*_CH_ from Watson et al.^38^

f Karplus-type relationships used in the simulations: ^3^*J*_CH_ from Tvaroška et al. (1989).^36^

g Karplus-type relationships used in the simulations: JCC/SU21.

h Experimental ^3^*J*_CH_ data: Tvaroška et al. (2002).^58^

i Experimental ^3^*J*_CH_ data: determined from β-d-Glc*p*6[^13^C]Me-OMe (**3*c***).

j Experimental ^3^*J*_CH_ data: from β-d-Glc*p*6Me-OMe (**3**).

### Conformational Analysis of Exo-Cyclic Torsions Using Limiting Values for ^3^*J*_H5,H6_ and ^3^*J*_H6,Me_

**Figure 5:**
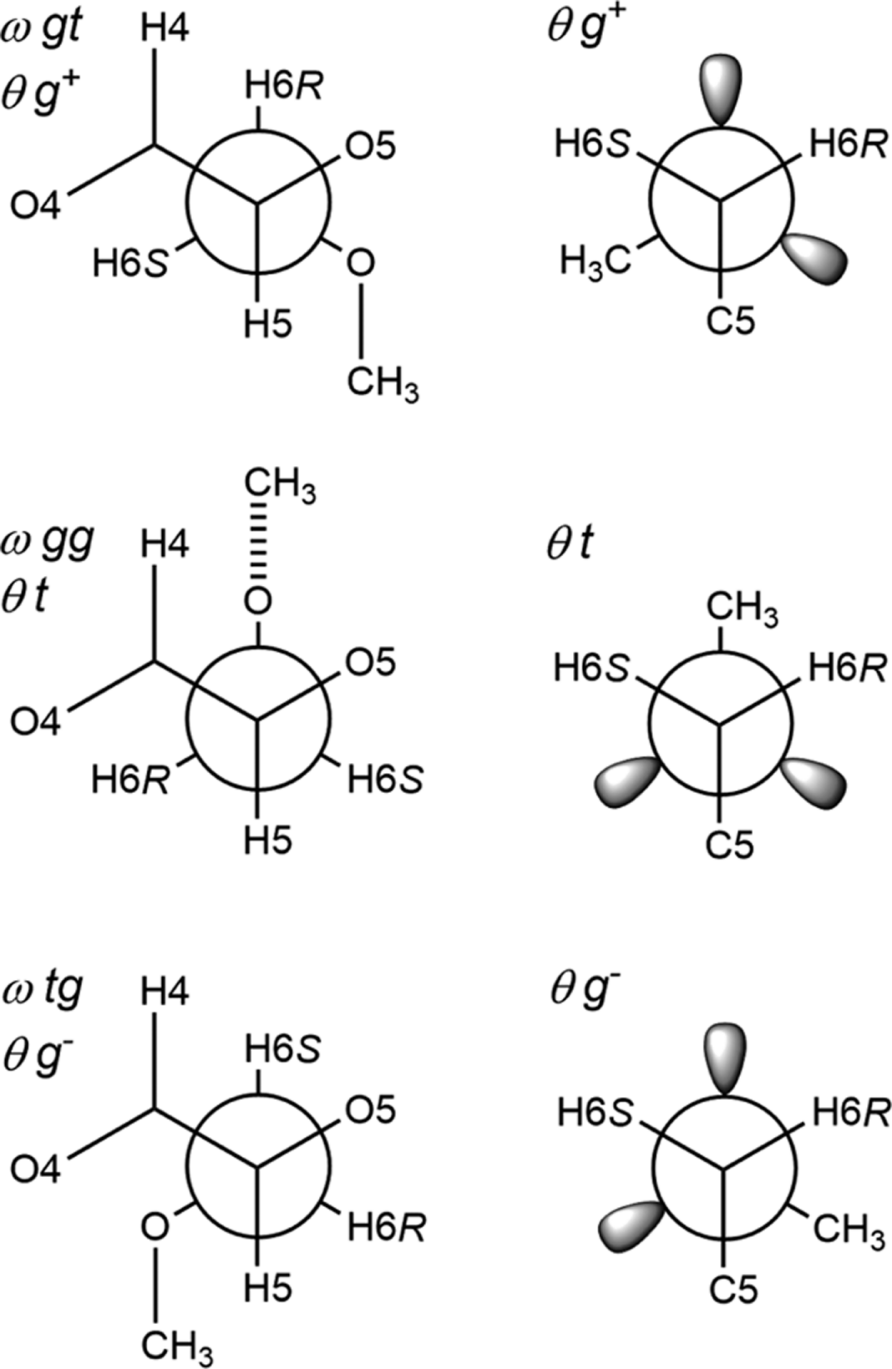
Newman projections along the C5–C6 bond in β-d-Glc*p*6Me-OMe (**3**). The staggered conformers are for the ω torsion angle (left side) referred to as *gt* (top), *gg* (middle), and *tg* (bottom) and the θ torsion angle is exemplified by *g*^+^ (top), *t* (middle), and *g*^–^ (bottom). The Newman projections along the C6–O6 bond for the θ torsion angle are given in an analogous way (right side).

The rotamer distribution at the ω and θ torsion angles (Figure 2) can be analyzed using a three-state model in which the limiting ^3^*J*_H5,H6_ and ^3^*J*_H6,Me_ values, respectively, of the conformational states are used together with the fact that the sum of populations for a given torsion is equal to unity. For the ω torsion angle, the limiting values are according to Stenutz et al.^33^ calculated for *gt* = +65°, *gg* = −65° and *tg* = 180° whereas Amarasekhara et al.^65^ utilized ideal staggered conformations. In deciding on a suitable Karplus-type relationship for the θ torsion angle for calculation of the limiting values with *g*^+^ = +60°, *g*^–^ = −60° and *t* = 180°, we relied on the recent results from DFT calculations of ^3^*J*_CH_ coupling constants by Li et al.,^66^ and choose the parametrization by Tvaroška et al.^36^ A schematic representation of the three conformational states of the ω torsion angle in conjunction with different states of the θ torsion angle is depicted in Figure 5. Whereas the parametrization by Stenutz et al.^33^ favors the *gt* conformational state over *gg*, that of Amarasekhara et al.^65^ results in a population where *gt* ≈ *gg* for β-d-Glc*p*-OMe (Table 2) using the herein collected NMR data, like previously observed. This is also the case for β-d-Glc*p*6Me-OMe, consistent with the results of Dahruman et al.^60^ who showed that the effect of the substituent group at O6 is small on the conformational distribution at the ω torsion angle. The error in the derived rotamer populations of the ω torsion angle is estimated to be <5%.

**Table 2:** Rotamer Populations for ω and θ Torsion Angles in β-d-Glc*p* (1), β-d-Glc*p*-OMe (**2**), and β-d-Glc*p*6Me-OMe (**3**) from MD Simulations and NMR Experiments

		ω rotamer distribution (%)		
compound	method	*gt*	*gg*	*tg*
**1**	MD	59	38	3
**2**	NMR^a^	55	36	9
	NMR^b^	51	49	0
	MD	65	32	3
**3**	NMR^a^	58	35	7
	NMR^b^	53	47	0
	MD	72	27	1

		θ rotamer distribution (%)		
compound	method	*g*^+^	*t*	*g*^–^
**1**	MD	30	43	27
**2**	MD	27	46	27
**3**	NMR^c^	30	50	20
	MD	18	78	4

a Limiting coupling constants from^a^ Stenutz et al.^33^

b Limiting coupling constants from Amarasekhara et al.^65^

c Limiting coupling constants from Tvaroška et al.^36^

Based on the limiting heteronuclear ^3^*J*_H6,Me_ values for β-d-Glc*p*6Me-OMe, the rotamer distribution of the θ torsion angle (Figure 5) was determined as *t* > *g*^+^ > *g*^–^ (Table 2). The error in the derived rotamer populations of the θ torsion angle is estimated to be <10%. In the case of a bulky O-tritylated group substituting position 6 of a glucosyl derivative, the *trans* conformation of the θ torsion angle was highly populated (88%).^33^ From DFT calculations on a pseudo-saccharide, it has been shown that ^2^*J*_H6*R*,H6*S*_ is considerably less negative when the torsion angle θ has an antiperiplanar arrangement (*t*), in contrast to when it is in either of the synclinal arrangements (*g*^+^ or *g*^–^). Whereas, ^2^*J*_H6*R*,H6*S*_ is highly dependent on the torsion angle θ, it varies to a smaller extent as a function of the ω torsion angle.^34^ Thus, that ^2^*J*_H6*R*,H6*S*_ is less negative in the 6-O-methylated derivative **3** compared to the unsubstituted analogue **2** (Table 1) is consistent with a significantly populated *t* state in β-d-Glc*p*6Me-OMe.

### Karplus-Type Equation for 6-O-Methylated Hexopyranoses

**Figure 6:**
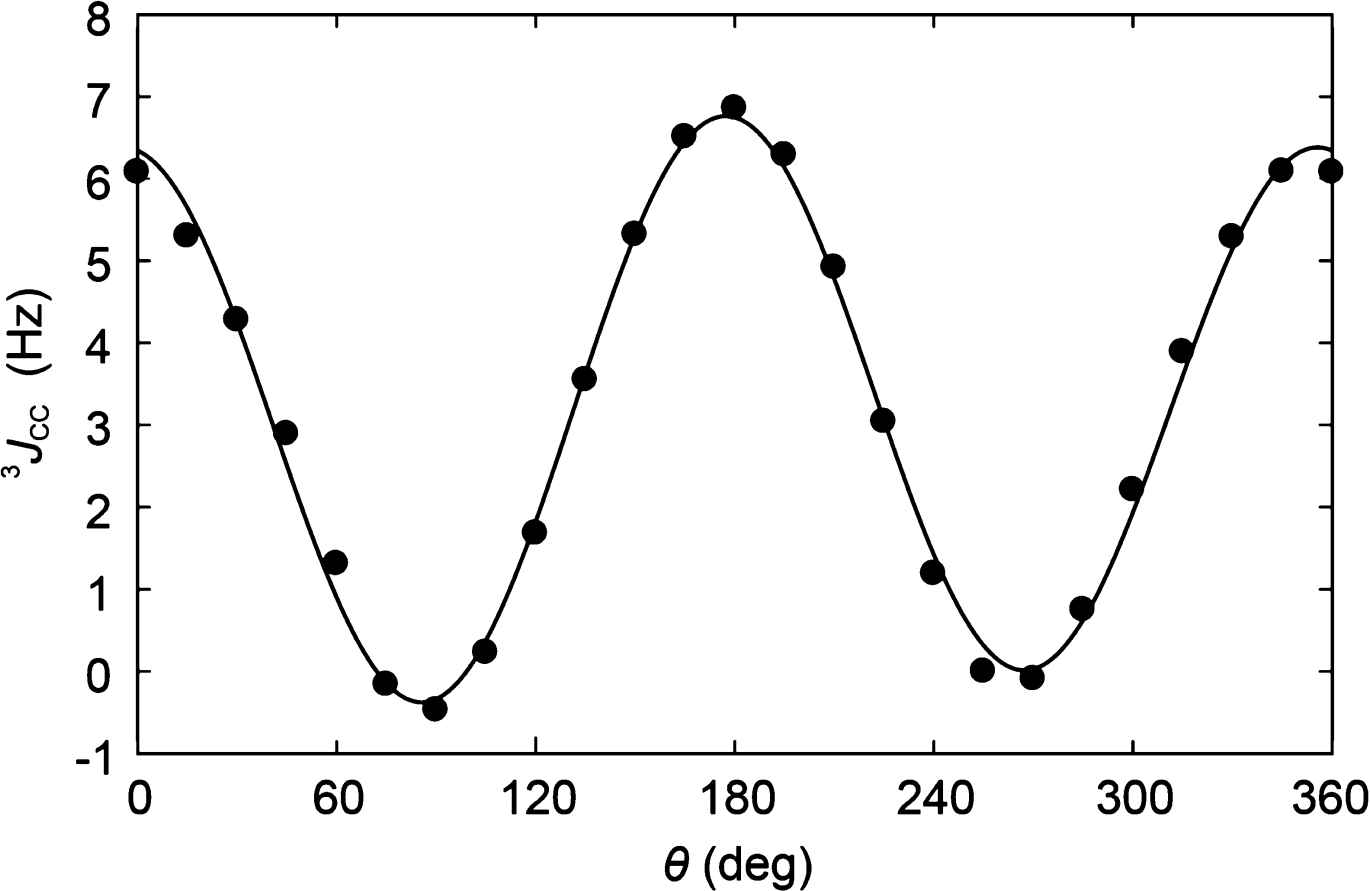
Karplus-type relationship (solid line) for the θ torsion angle in compound **3** derived from calculated ^3^*J*_COCC_ values (filled circles).

Calculations of NMR spin–spin coupling constants by theoretical methods are highly valuable in conformational analysis of saccharides^17,67^ and can form the basis for developing and/or improving Karplus-type relationships that subsequently can be used on their own in studies of oligo- and polysaccharide conformation and dynamics. To reduce the complexity and to increase the computational efficiency, pseudo-saccharides devoid of hydroxyl groups can be utilized in quantum mechanical calculations of conformational preferences^68^ and such a model (compound **4**) was available from the literature for which ^3^*J*_COCC_ coupling constants had been calculated as a function of torsion angle rotation.^69^ Based on these computed data, we herein propose a Karplus-type relationship denoted JCC/SU21 given by
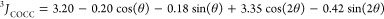
13related to the C5–C6–O6–C_Me_ torsion angle θ (Figure 6). In comparison to the Karplus-type relationship given for methoxyethane that has its maximum at 0° as presented by Hadad et al.,^17^ one can note that JCC/SU21 instead has its maximum at ∼180°, with a minute phase shift of the curve of only −4°, as the electronegative ring oxygen is a terminal substituent to the coupling path.^69^ With the homonuclear ^3^*J*_C5,Me_ coupling constant determined from β-d-Glc*p*6[^13^C]Me-OMe and a pertinent Karplus-type relationship implemented, conformational analysis can be extended further in conjunction with MD simulations.

### Molecular Dynamics Simulations of Monosaccharides with Comparison to Experiments

**Figure 7:**
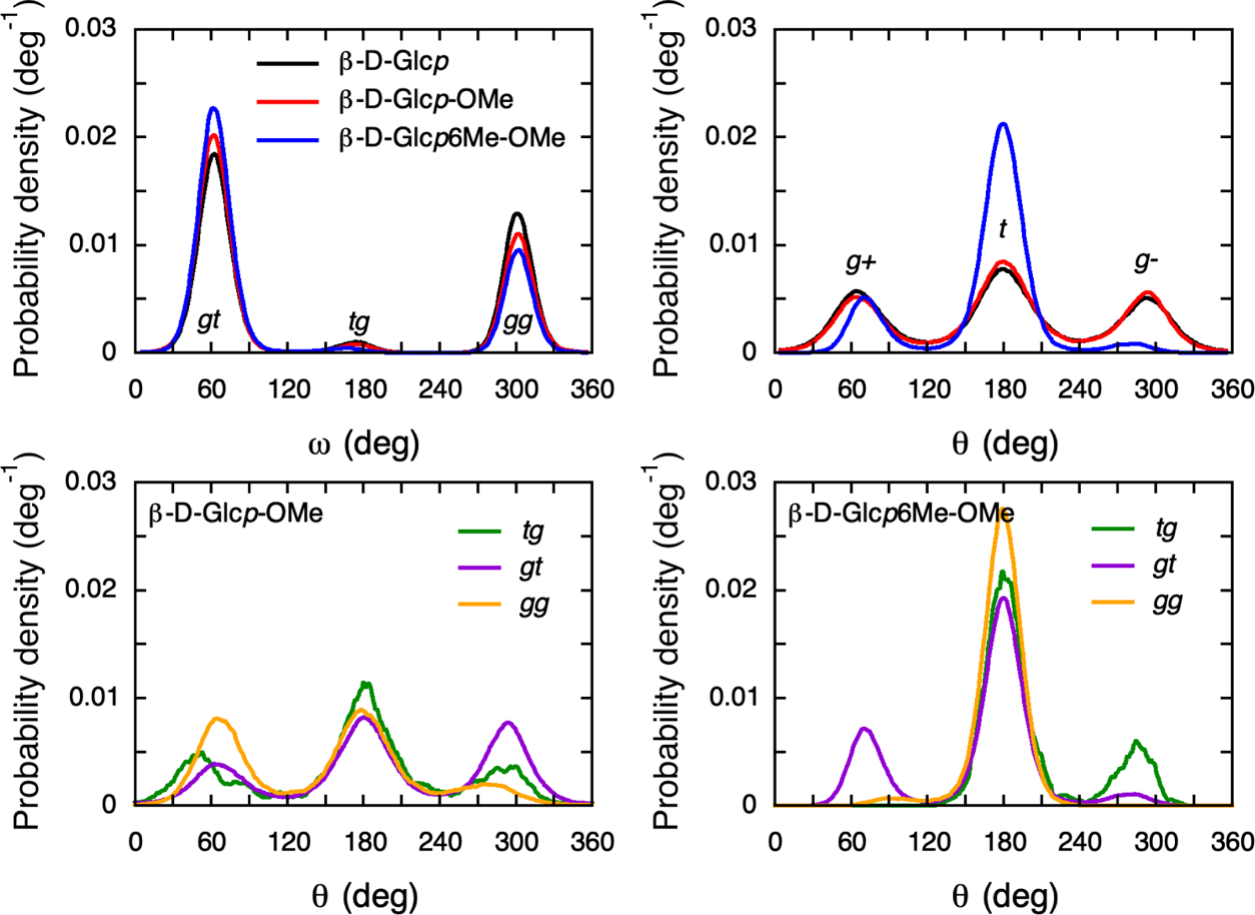
Top row: Distributions of the torsion angles ω (left) and θ (right) for the three monosaccharides from simulations at 298 K. Bottom row: Distributions of θ extracted from compounds **2** (left) and **3** (right) with ω in either of the three states: *tg*, *gt*, or *gg*.

MD simulations were performed of compounds **1** to **3** in water, and the rotamer distributions of ω and θ were sampled (Figure 7). The simulations show that both ω and θ are affected by methylation. For ω, the population in *gt* increases slightly from glucopyranose to the methyl glucopyranoside, and then further upon 6-O-methylation, in both cases at the expense of *gg*. The population in *tg* remains small and relatively unaffected. Although the differences are small, this trend is supported also by calculated populations from NMR (Table 2) with respect to the difference between compounds **2** and **3**.

The simulated distributions for θ are almost identical for compounds **1** and **2**, displaying a close to even distribution between the three conformational states with a small predominance for *trans* conformation. This implies that an O-methyl group at C1 does not affect the rotation of the hydroxyl group at C6. With O-methylation in position six, the population in *gauche*^–^ almost vanishes completely, and the *trans* conformer becomes the dominating one. This trend is again supported by the experiments (Table 2) although the effect is not as dramatic.

The rotamer states of ω and θ in glucose-based monosaccharides were previously shown to be correlated.^34^ For that reason, distributions of θ in compounds **2** and **3** were calculated separately for the cases of having ω in either *tg*, *gt*, or *gg* (Figure 7). Interestingly, θ depends weakly on ω in the non-O6-methylated compound, where the *gt* and *gg* conformations favor having θ in *g*– and *g*+, respectively. With a methyl group on O6, the correlation becomes stronger. Specifically, although *trans* dominates for θ in all cases, with ω in *gg*, only the *trans* conformation is permitted.

**Figure 8:**
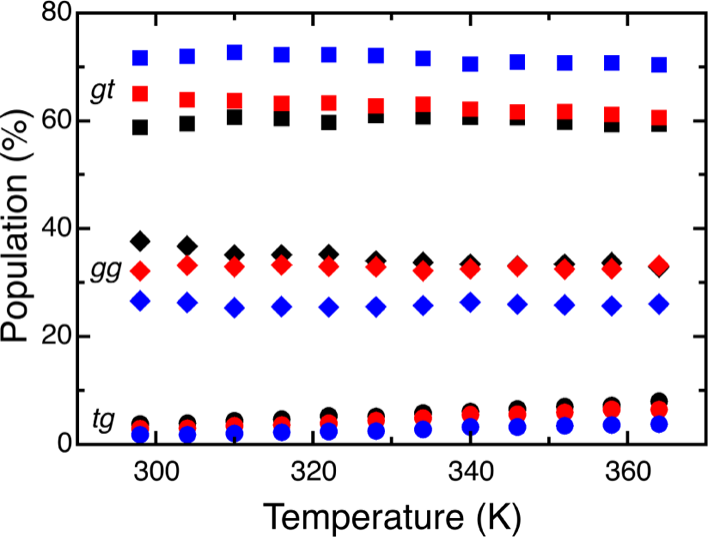
Populations of ω rotamers: *tg* (circles), *gg* (diamonds), and *gt* (squares), as a function of temperature for the three monosaccharides: β-d-Glc*p* (black), β-d-Glc*p*-OMe (red), and β-d-Glc*p*6Me-OMe (blue).

The population distributions for ω at different temperatures were also calculated from the simulations (Figure 8). The populations show only weak dependence on temperature, which is in line with previous simulation results on both methylated and nonmethylated saccharides.^21,40,70^ Interestingly, the small difference between compounds **1** and **2** vanishes at increased temperatures, but the difference to compound **3** remains.

From the simulated distributions, NMR coupling constants for compounds **2** and **3** were calculated using the appropriate Karplus-type relationships. The overall agreement between experimentally determined *J* coupling constants is good (Table 1), although an even better agreement would require additional fine-tuning of the GROMOS 56 carbohydrate force field and as such could reveal the Karplus equations to be used preferably. By an NMR experiment based on heteronuclear ^3^*J*_H6,Me_ couplings, the *trans* conformational state for the θ torsion angle of compound **3** was determined to be the major one, which indeed was the case also in the MD simulations. However, simulation overestimates this conformational preference somewhat, furthermore stressed by the computed homonuclear ^3^*J*_C5,Me_ coupling, which is larger than determined from experiment. This observation supports the fact that the herein proposed Karplus-type relationship for ^3^*J*_COCC_ (Figure 6 and eq 13) referred to as 3JCC/SU21 will be a useful complement in the conformational analysis of oligo- and polysaccharides.

### Solution Thermodynamics of Methylated Monosaccharides from Simulations

In addition to a solution structure, it is interesting to investigate whether the model can capture relevant solvation thermodynamics of the model compounds. Experimental data is scarce, but Jasra and Ahluwalia^71^ have reported experimental partial molar volumes (*V*_2_^0^) for β-d-Glc*p* and β-d-Glc*p*-OMe at infinite dilution, which shows the effect of methylation at O1. Partial molar volumes were thus calculated from long equilibrium simulations of a single sugar molecule in water and compared to simulations of pure water. Simulated values for *V*_2_^0^ are high compared to experimental data (Table 3), and the volume per glucose molecule is more than 13% higher in the simulations. By comparing *V*_2_^0^ for both methylated and non-methylated sugars, the change in *V*_2_^0^ resulting from methylation, Δ*V*_2_^0^, is calculated. The calculated increase, Δ*V*_2_^0^, for the 1-O-methyl substitution in simulations is close to the experimental value in absolute numbers, but still 16% higher in relative terms. For the 6-O-methylation, for which there is no experimental result, the calculated Δ*V*_2_^0^ is higher than for methylation at O1. This is likely a reflection of the higher degree of hydration of the hydroxyl group at C6 than of the one at C1, as a methyoxy group constitutes a larger perturbation to the solvent.

**Table 3:** Partial Molar Volumes at Infinite Dilution in cm^3^ mol^–1^

	*V*_2_^0^ (sim^a^)	Δ*V*_2_^0^ (sim)	*V*_2_^0^ (expt^b^)	Δ*V*_2_^0^ (expt^b^)
β-d-Glc*p*	126.7		111.7	
β-d-Glc*p*-OMe	144.3	17.6	132.1	20.4
β-d-Glc*p*6Me-OMe	163.7	19.4		

a Statistical error is on the order of 0.5 cm^3^ mol^–1^.

b Experimental values from Jasra and Ahluwalia.^71^

The free energy of solvation Δ*G*_s_ was computed by decoupling all solute–solvent interactions, as described in the Methods section, for the three monosaccharides in water (Table 4). The computed Δ*G*_s_ values are large and negative, indicating a strong preference for the solvated state over the vapor phase, as expected. The calculated Δ*G*_s_ for β-d-Glc*p*, −68.0 kJ mol^–1^, compare reasonably well to other values found in the literature. Klimovich and Mobley calculated it to −71 kJ mol^–1^ using the General Amber Force Field,^72^ and López et al. obtained −89 kJ mol^–1^ using GROMOS 45.^73^ The simulations furthermore show a clear effect of methylation: Δ*G*_s_ increases by more than 10 kJ mol^–1^ as a result of having an additional methyl group in both the O1 and O6 positions. This is a direct consequence of replacing polar hydroxyl groups with non-polar methyl groups, which makes the solutes considerably less hydrophilic.

**Table 4:** Calculated and Experimental Solvation Free Energies Δ*G*_s_ in kJ mol^–1^

	MeOH	DME	β-d-Glc*p*	β-d-Glc*p*-OMe	β-d-Glc*p*6Me-OMe
Δ*G*_s_^calc^	–17.7 ± 0.1	–2.64 ± 0.1	–68.0 ± 0.2	–54.7 ± 0.1	–44.5 ± 0.2
Δ*G*_s_^expt^	–21.32^a^	–7.94^a^	–106.8 ± 0.9^b^		

a From ref (77).

b From ref (75) (for α-d-Glc*p*).

Solvation free energies of large nonvolatile solutes such as monosaccharides are not trivial to determine experimentally. For d-glucose, experimental vapor pressures are available,^74^ which together with its molar solubility in water can be used to calculate the solvation free energy. Based on experimental data, Δ*G*_s_ for d-Glc*p* becomes −106.8 kJ mol^–1^, which is considerably larger than the values from the present simulations of monosaccharides. In fact, the solvation free energy of sugars is notoriously difficult to predict using computational methods: in the *SAMPL2 blind prediction challenge*(75) half of the contributions (using a wide array of different models) missed the experimental Δ*G*_s_ for d-glucose by more than 25 kJ mol^–1^. The relative complexity and flexibility of the sugar molecules leading to difficulties to achieve converged sampling were mentioned as a likely cause, although here, with the use of REMD and long simulations this is less probable. A more plausible explanation in our opinion is the inherent inaccuracy of the force field parameters which can have dramatic effects on solvation-free energies. Some empirical force fields have been shown to systematically underestimate the solvation-free energy of sugars and other hydroxyl-rich compounds, presumably due to poor optimization of alcohols.^72^ Furthermore, it has been shown that depending on which charge equilibration scheme was used in the model development, the calculated solvation free energy of glucose could differ by as much as 60 kJ mol^–1^.^76^

However, despite difficulties to reproduce absolute solvation free energies simulations can still be useful. The peculiar phase behavior of hydrated MC likely depends on a delicate balance between the solvation energies of its methylated and non-methylated parts. Therefore, it is more important that the model replicates *differences* in solvation free energy, ΔΔ*G*_s_, between the methylated and non-methylated model compounds. However, because, to the best of our knowledge, no experimental hydration-free energies for the methylated sugars are available, a slightly different approach is taken which is described in the next section.

### Free Energy of Solvation of Small Model Compounds

In order to validate the free energy difference between hydroxyl and O-methyl groups, simulations were performed using small model compounds. The test solutes opted for were methanol (MeOH) and DME, which differ in that the OH group in MeOH is substituted for an O-methyl group. Importantly, the calculated values can be compared to available experimental data for the hydration-free energies, which can be derived from tabulated Henry’s law constants at infinite dilution.^77^

The calculated values of Δ*G*_s_ for both MeOH and DME are too small compared to experimental data, differing by 3.6 and 5.3 kJ mol^–1^, respectively (Table 4). This is in line with that Δ*G*_s_ for the sugars were also underestimated, although the fact that the error is larger for DME shows that it is not only the OH groups that are to blame. On the other hand, the difference between the two, ΔΔ*G*_s_, which is of greater importance for the present work is slightly overestimated by the simulations (Table 5) and differs by only 1.7 kJ mol^–1^ from the experimental value. This is reassuring because it suggests that the present parameters correctly reproduce both the sign and, within reasonable accuracy, the magnitude of the change in solvation free energy when hydroxyl groups are transformed into O-methyl groups, also in more complex compounds.

**Table 5:** Differences in Solvation Free Energy ΔΔ*G*_s_ in kJ mol^–1^

	MeOH/DME	β-d-Glc*p*-OMe/β-d-Glc*p*6Me-OMe
experimental	13.38	
solvation	15.1 ± 0.2	10.2 ± 0.3
mutation: Δ*G*_mut_^wat^ – Δ*G*_mut_^vac^	13.9 ± 0.1	10.6 ± 0.1

### Free Energy of Solvation from Computational Alchemy

It is not obvious that properties for monosaccharides automatically can be extrapolated to oligo- or polysaccharides, such as cellulose, and for that reason, calculations on larger solutes were undertaken. However, the method used above is not well suited for computing solvation-free energies of complex molecules such as oligosaccharides, due to the long simulations needed to ensure convergence. Here, we take a slightly different approach and consider the change in free energy of transforming the non-methylated oligosaccharide into its methylated counterpart using computational alchemy (see Methods). Because this transformation constitutes a much smaller change than decoupling the whole oligosaccharide from the solvent, much more rapid convergence can be expected. It can be noted that this is similar to the approach taken by Yu et al.^24^ where the mutations were treated as single-step perturbations, that is, without intermediate states in λ.

To validate our approach, the alchemical transformations were performed on both monosaccharides and on MeOH for which the Δ*G*_s_ was already calculated. In this way, all paths in the thermodynamic cycle can be independently assessed, and the internal consistency can be checked. For the case of the transformation of MeOH into DME, ΔΔ*G*_s_ was 1.2 kJ mol^–1^ smaller when calculated from the mutations compared to the full decoupling simulations. This value was thus closer to the experimental result, differing by as little as 0.5 kJ mol^–1^. For the case of monosaccharides, the difference between the alchemical transformation and the decoupling was even smaller, only 0.4 kJ mol^–1^ (Table 5), indicating good internal consistency.

Thus, although absolute solvation free energies are not particularly well reproduced by simulations, we conclude that the present parameters reproduce the free energy change of methylation with sufficient accuracy, and further that the considerably less computationally expensive method of alchemical mutation of hydroxyl groups reflects that change in a satisfactory manner.

### Simulation of Methylated Cellooligomers

The alchemical approach of mutating hydroxyl groups into methyl groups was applied to aqueous cellotetraose, as a model for longer glucan chains. In the common synthesis route for MC, glucan chains are heterogeneously 2-, 3-, and 6-O-methylated. Here, one of the middle residues in cellotetraose was individually methylated in either of the three positions, respectively. The free energy change associated with the mutation depends on in which position it occurs. The calculated ΔΔ*G*_s_ was smallest for 3-O-methylation (2.3 ± 0.2 kJ mol^–1^), followed 2-O-methylation (3.6 ± 0.2 kJ mol^–1^), which both were considerably smaller than for 6-O-methylation (14.6 ± 0.2 kJ mol^–1^). Note that this order should not be confused with reactivities, as the calculated energy reflects the change in solvation properties, which are consequences of the methylation, not the chemical reaction itself. In fact, experiments show that O2 is the one most prone to methylation in both glucose derivatives^78,79^ and in cellulose under homogenous^14^ reaction conditions, whereas the reactivities of O2 and O6 are similar under heterogeneous reaction conditions.^80^

**Figure 9:**
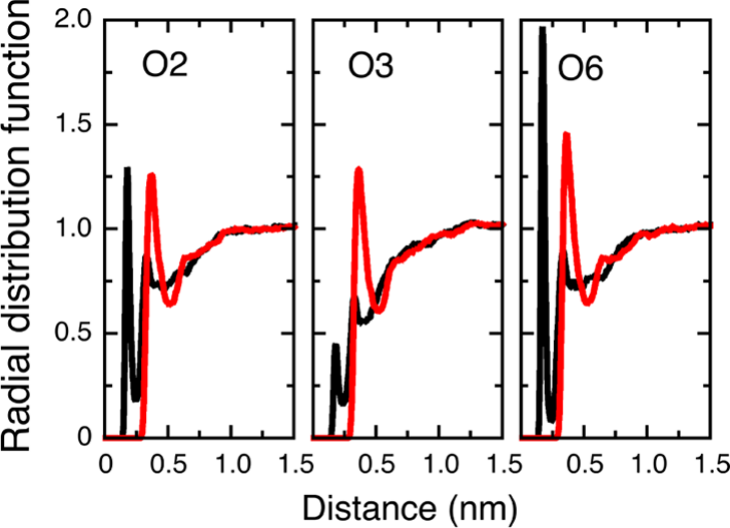
Radial distribution functions between hydroxyl proton (black) or methyl group (red) to oxygen atoms of water, for O2, O3, and O6 in the second glucose unit of cellotetraose, calculated from 5 ns of the simulations at 298 K (employing REMD).

The results presented here stand in sharp contrast to Yu et al.^24^ who calculated the average ΔΔ*G*_s_ to 43 kJ mol^–1^, 24 kJ mol^–1^, and 17 kJ mol^–1^, respectively, for 2-, 3-, and 6-O-methylation in cellononaose using an early version of the GROMOS carbohydrate force field. The result for C6 is similar to our result, but for C2 and C3, the value is larger and the ordering of the respective positions different than in the present work. The most likely explanation for this discrepancy is effects of poor convergence. While the previous result was based on five-ns simulations at room temperature, our results are based on considerably more data and also employed REMD, which has been shown before to be essential for satisfactory convergence of the conformational dynamics of carbohydrates.^40,81^ Moreover, we argue that the order O6 > O2 > O3 is quite reasonable. Radial distribution functions (RDF) between hydroxyl protons (for the unsubstituted case) or methyl groups (for the methylated case), and oxygen atoms of water, for O2, O3, and O6, respectively, are shown in Figure 9. It is evident from the RDFs that the water structure around the methyl groups is very similar in all three positions. For the unsubstituted cellotetraose, on the other hand, there is clear difference visible in the first and second solvation peaks. Their respective heights follow the same order as the corresponding ΔΔ*G*_s_. This points to a correlation between free energy of solvation and magnitude of perturbation of the water structure: the more hydrated the hydroxyl group, the larger the perturbation from methylation, which manifests in a larger ΔΔ*G*_s_.

**Figure 10:**
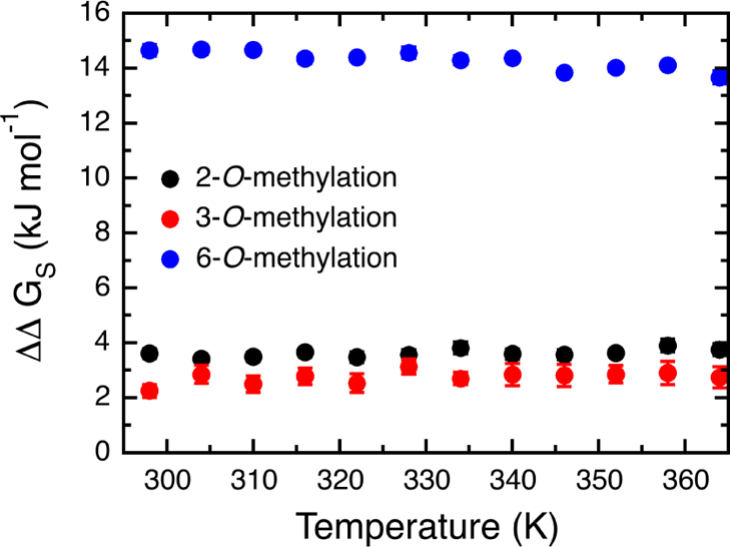
Change in solvation free energy upon methylation of the second glucose unit in cellotetraose in the O2, O3, and O6 positions, respectively, as a function of temperature.

Furthermore, we note that ΔΔ*G*_s_ exhibits only a very weak dependence on temperature, if any (Figure 10), for all three positions. Thus, the present calculations are not sufficient to explain the more complex temperature behavior of MC suspensions.

### Effect on Flexibility

**Figure 11:**
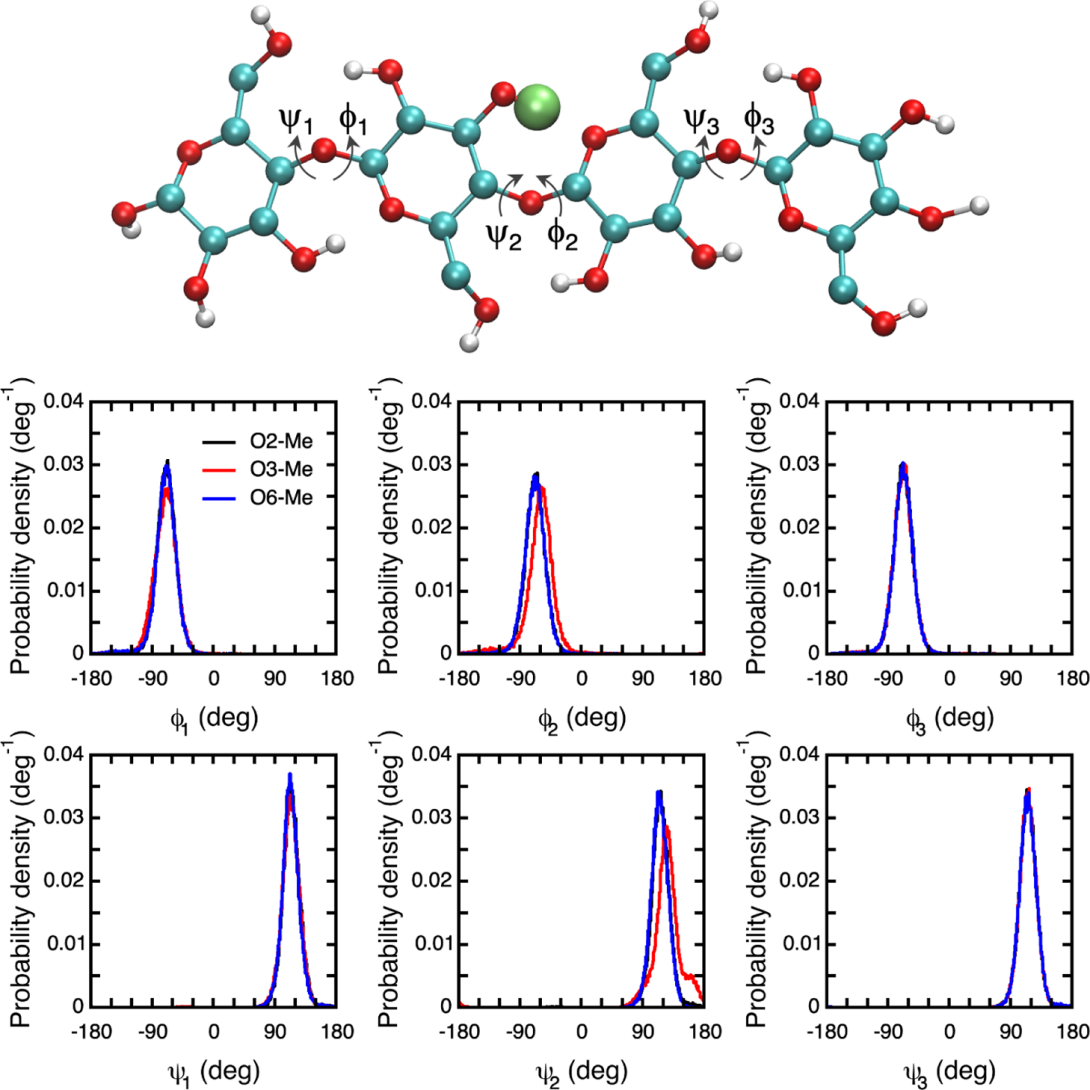
Cellotetraose, O3-methylated in the 2nd residue, and the torsion angles (IUPAC nomenclature) of the glycosidic linkages. The size of the methyl group has been exaggerated (top). Probability distributions of ϕ and ψ for the glycosidic linkages in the methylated tetrasaccharides (bottom).

It is also of interest to investigate whether methylation affects the conformation of polysaccharides in solution, in part because that can affect the solubility. The large-scale conformation of polysaccharides is governed by the local conformations of the glycosidic linkages, which can be described using two torsion angles, ϕ and ψ. To that end, distributions of these torsion angles were calculated from the simulations of the methylated cellotetraose (Figure 11). The distributions show that it is only 3-O-methylation that affects the glycosidic linkage, and only the linkage that is the closest to the substitution point, ϕ_2_ and ψ_2_ in the present case. Both ϕ_2_ and ψ_2_ are shifted to higher torsion angles indicating a slightly larger twist between two consecutive units, and the distribution of ψ_2_ is further broadened indicating slightly higher flexibility. However, these changes are quite small and lead to no significant deviation from an extended conformation.

Hydrogen bonding has been suggested to affect the conformation. A cellobiose unit in its most probable conformation can simultaneously form two *trans*-glycosidic hydrogen bonds between O3H···O5′ and O6H···O2H′. Methylation thus removes these hydrogen bonding possibilities, and this has been suggested to lead to increased flexibility.^25^ However, MD simulations showed that the ϕ/ψ space for disaccharides of mannose and allose, where *trans*-glycosidic hydrogen bonding is suppressed due to having the hydroxyl groups in axial orientation, is almost identical to cellobiose.^82^ This means that the difference observed from methylation at O3 probably is a consequence of increased steric interactions.

The observations presented herein agree with earlier simulations of methylated cellobiose in water using the same force field,^21^ which also noted an increase in ϕ and ψ upon 3-O-methylation. However, in a study of methylated cellononaose in water^24^ using the same force field, a slight decrease of both ϕ and ψ was observed. Interestingly, it was also found in a more recent study employing the GLYCAM06 force field that blocks of successive O3 methylation resulted in a significant decrease in ψ, compared to a substitution pattern where every other unit was methylated.^25^ This indicates an effect from substitution pattern on conformational flexibility. It should be noted though that the latter simulations may not necessarily be well-converged. It is well known that conformations of saccharides, both hydroxymethyl rotations^40^ and glycosidic linkages,^83^ take a long time to converge to their equilibrium distributions in water at room temperature, unless methods to enhance the sampling are employed. This complicates the comparison between simulation studies and may account for the discrepancies.

## Conclusions

The present study reports on both conformational and solvation properties of methylated mono- and tetrasaccharides from solution NMR and molecular dynamics simulations. The simulations show overall good agreement with experimental NMR coupling constants and derived populations relevant to the ω torsion that describes the orientation of the primary alcohol. It is found that 6-O-methylation affects ω only slightly, both as determined from NMR experiment and when compared among the MD simulations of the monosaccharides. The NMR experiment reveals the population preference of the θ torsion angle of β-d-Glc*p*6Me-OMe as *t* > *g*^+^ > *g*^–^, captured also by the MD simulation, albeit with the *trans* state overestimated.

Calculated differences in hydration free energies between non-methylated and methylated molecules using two different methods—either by decoupling the solute–solvent interactions or by mutating the hydroxyl group into an O-methyl group using computational alchemy—yield internally consistent results. Moreover, when these methods are applied to the difference between methanol and DME, the calculated free energy difference is also consistent with experimental data obtained from Henry’s law constants at infinite dilution, providing a general validation of both the method and the potential parameters.

The computational alchemy approach was subsequently applied to methylated cellotetraose as a model for MC. It was found that methylation increases the hydration free energy by 2.3, 3.6, and 14.6 kJ mol^–1^ for 3-, 2-, and 6-O-methylation, respectively. These results stand in contrast to those previously reported, which highlights the need for applying both enhanced sampling methods and long simulations to obtain converged results for carbohydrates in solution.

The results presented herein have led to insights about the Karplus equations and their relationships to MD simulations, in particular the GROMOS 56 carbohydrate force field, and will serve as an important basis for simulation studies on more specialized topics related to MC and other methylated carbohydrates.

## Supporting Information Available

Description of the chemical synthesis of model compounds; ^1^H and ^13^C NMR spectra of monosaccharides; and plots of all Karplus-type equations (PDF)
