# Supplementary material for: O-Methylation in Carbohydrates: An NMR and MD Simulation Study with Application to Methylcellulose
Source: J Phys Chem B. 2021 Oct 27;125(43):11967–79. doi: 10.1021/acs.jpcb.1c07293 (PMC8573740; doi:10.1021/acs.jpcb.1c07293)
Supplement: Supplementary file 1 — jp1c07293_si_001.pdf [file jp1c07293_si_001.pdf]

O-Methylation in Carbohydrates: an NMR and MD Simulation Study with Application to Methylcellulose

Alessandro Ruda[1], Göran Widmalm[1], and Jakob Wohlert[2,3]

[1] Department of Organic Chemistry, Arrhenius Laboratory, Stockholm University, SE-106 91 Stockholm, Sweden

[2] Department of Fiber and Polymer Technology, School of Chemistry, Biotechnology and Health, KTH Royal Institute of Technology, SE-100 44 Stockholm, Sweden

[3] Wallenberg Wood Science Center, KTH Royal Institute of Technology, SE-100 44 Stockholm, Sweden

Supporting Information

Table of Contents

Chemical synthesis of monosaccharides..S2 – S5

^{1}H and ^{13}C NMR spectra of monosaccharides...S6 – S10

Karplus-type relationships...S11 – S12

General methods. Dry acetonitrile (ACN) was purchased from Honeywell Research Chemicals (Bucharest, Romania), and dry *N*,*N*-dimethylformamide (DMF) and dichloromethane (DCM) were purchased from Acros Organics (Morris Plains, NJ, USA). Methanol (MeOH) was dried over 4 Å molecular sieves. Powdered molecular sieves (4 Å) were activated by heating under high vacuum. All reagents were purchased from Sigma-Aldrich (Geel, Belgium) unless otherwise specified and were used as received. A nitrogen flow was used for reactions requiring inert atmosphere. TLC was carried out on silica gel 60 F254 plates (20 × 20 cm, 0.2 mm thickness) and monitored with UV light (254 − 360 nm) or by a staining solution prepared from ceric ammonium sulfate (2 g) in ethanol (40 mL) and 2 M sulfuric acid (40 mL). Column chromatography was performed on a Biotage Isolera flash chromatography system (Uppsala, Sweden) using KP-Sil or HP-Sil snap silica gel cartridges and purification on Sep-Pak C18 Plus short cartridges, 55 – 105 µm particle size (Waters, Milford, MA, USA). MQ water was obtained from an Elga Purelab Ultra Genetic Water Purification System (High Wycombe, UK).

One-dimensional ^{1}H and ^{13}C, 2D ^{1}H,^{1}H-COSY and multiplicity-edited ^{1}H,^{13}C-HSQC NMR experiments for the characterization of compounds were recorded in $CDCl_3$ or D_2O at 298 K on a Bruker AVANCE III HD 400 MHz spectrometer equipped with a 5 mm BBF/H/D 5.0 Z probe or a Bruker AVANCE III 600 MHz spectrometer equipped with a 5 mm TXI inverse Z-Gradient ^{1}H/^{31}P/^{13}C probe, respectively. NMR chemical shifts were referenced for protected compounds to internal tetramethylsilane (δ_H 0.0) and for ^{13}C to the $CDCl_3$ solvent peak (δ_C 77.36); for deprotected compounds internal sodium 3-trimethylsilyl-(2,2,3,3-$^{2}H_4$)-propanoate in D_2O (δ_H 0.0) and external 10% dioxane in D_2O (δ_C 67.4) were used as chemical shift references.

High-resolution mass spectrometry (HRMS) experiments were conducted on Bruker Daltonics micrOTOF or micrOTOFQ spectrometers (Bremen, Germany) using electrospray ionization (ESI) in the positive mode. Samples were prepared using a solution of acetone and H_2O in a 1:1 ratio.

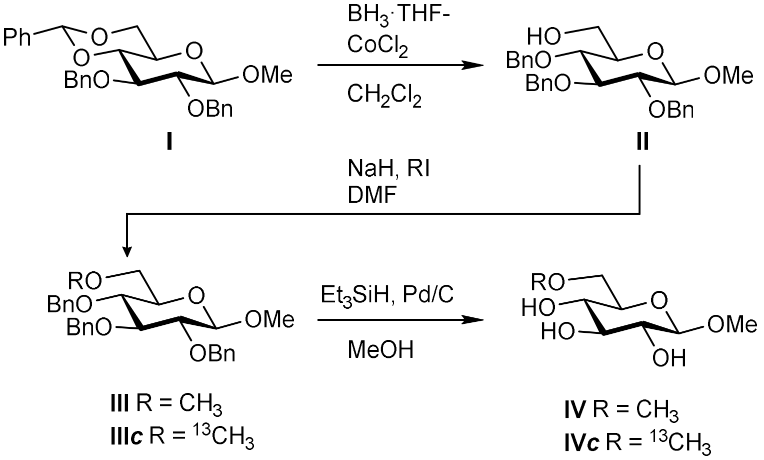

Scheme S1. Synthesis of methyl 6-*O*-methyl-β-D-glucopyranoside.

Methyl 2,3,4-tri-*O*-benzyl-β-D-glucopyranoside (II)

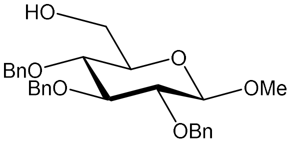

$C_{28}H_{32}O_6$
464.56 g/mol

To a stirred 1 M BH_3•THF solution (2.6 mL – 3 eq) at r.t. containing methyl 2,3-di-*O*-benzyl-4,6-*O*-benzylidene-β-D-glucopyranoside (**I**, 0.86 mmol – 1 eq) $CoCl_2$ (2.58 mmol – 3 eq) was added portionwise whereafter stirring continued for 5 min. The mixture was then diluted with EtOAc, filtrated and aq $NaBH_4$ (0.5 eq) was added. The organic fraction was washed with aq $NaHCO_3$ solution, water, dried over Na_2SO_4 and concentrated under vacuum to afford a yellow oil. Purification by flash column chromatography (R_f: 0.55, dichloromethane/ethyl acetate, 9:1) gave a white powder (85% yield). ^{1}H NMR (400 MHz, $CDCl_3$): δ 7.36 – 7.25 (*m*, 15H, Ar (Bn)), 4.95 – 4.62 (*m*, 6H, CH_2 (Bn)), 4.35 (*d*, 1H, $J_{H1,H2}$ = 7.80 Hz, H-1), 3.88 (*ddd*, 1H, $J_{H5,H6a}$ = 2.75 Hz, $J_{H6a,H6b}$ = 11.88 Hz, $J_{H6a,OH}$ = 5.89 Hz, H-6a), 3.72 (*ddd*, 1H, $J_{H5,H6b}$ = 4.54 Hz, $J_{H6a,H6b}$ = 11.88 Hz, $J_{H6b,OH}$ = 7.56 Hz, H-6b), 3.67 (*dd*, 1H, $J_{H2,H3}$ = 9.01 Hz, $J_{H3,H4}$ = 9.00, H-3), 3.57 (*dd*, 1H, $J_{H3,H4}$ = 9.00, $J_{H4,H5}$ = 9.62 Hz, H-4), 3.57 (*s*, 3H, CH_3), 3.39 (*dd*, 1H, $J_{H1,H2}$ = 7.80 Hz, $J_{H2,H3}$ = 9.01 Hz, H-2), 3.37 (*ddd*, 1H, $J_{H4,H5}$ = 9.62 Hz, $J_{H5,H6a}$ = 2.75 Hz, $J_{H5,H6b}$ = 4.54 Hz, H-5), 1.89 (*dd*, $J_{H6a,OH}$ = 5.89 Hz, $J_{H6b,OH}$ = 7.56 Hz, 1H, OH). ^{13}C-NMR (100 MHz, $CDCl_3$): δ 138.8, 138.7, 138.3, 128.8, 128.7, 128.4, 128.23, 128.18, 128.0, 127.9 (Ar), 105.1 (C1), 84.7 (C3), 82.7 (C2), 77.8 (C4), 76.0 (CH_2), 75.4 (CH_2), 75.3 (C5), 75.1 (CH_2), 62.3 (C6), 57.6 (CH_3). ESI-HRMS: $[M + Na]^+$ *m/z* calculated for $C_{28}H_{32}O_6Na$ 487.2091, found 487.2102 (error −1.1 ppm).

Methyl 2,3,4-tri-*O*-benzyl-6-*O*-methyl-β-D-glucopyranoside (III)

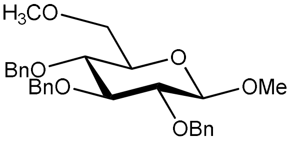

$C_{29}H_{34}O_6$
478.59 g/mol

To a solution of methyl 2,3,4-tri-*O*-benzyl-β-D-glucopyranoside (0.52 mmol – 1 eq) in dry DMF (5 mL) NaH (1.3 mmol – 2.5 eq) was added at 0 °C under N_2 atmosphere and the mixture was left under stirring for 2 h. MeI was hence added dropwise (1.04 mmol – 2.0 eq) and stirring continued for 6 h. The reaction was quenched by addition of MeOH, and the mixture was co-evaporated with toluene, diluted with EtOAc and washed with H_2O and brine. The organic fraction was dried over Na_2SO_4 and concentrated under vacuum to afford a yellow oil. The product was purified by flash column chromatography (R_f: 0.43, petroleum ether/ethyl acetate, 8:2) to yield a colorless oil (75% yield). ^{1}H NMR (400 MHz, $CDCl_3$): δ 7.37 – 7.22 (*m*, 15H, Ar (Bn)), 4.96 – 4.57

(*m*, 6H, CH_2 (Bn)), 4.30 (*d*, 1H, $J_{H1,H2}$ = 7.80, H-1), 3.66 – 3.56 (*nr*, 2H, H-6a, H6b), 3.64 (*nr*, 1H, H-3), 3.60 (*nr*, 1H, H-4), 3.55 (*s*, 3H, CH_3), 3.44 (*dd*, 1H, $J_{H1,H2}$ = 7.80, $J_{H2,H3}$ = 8.65, H-2), 3.41 (*m*, 1H, J_{HH} = 2.90, J_{HH} = 4.30, H-5), 3.36 (*s*, 3H, 6-*O*-CH_3). ^{13}C NMR (100 MHz, $CDCl_3$): δ 138.9, 138.8, 138.5, 128.6, 128.3, 128.2, 128.03, 127.98, 127.8, 127.7 (Ar), 105.0 (C1), 84.8 (C3), 82.5 (C2), 77.9 (C4), 75.8 (CH_2), 75.2 (CH_2), 74.93 (CH_2), 74.86 (C5), 71.4 (C6), 59.6 (6-*O*-CH_3), 57.3 (CH_3). ESI-HRMS: $[M + Na]^+$ *m/z* calculated for $C_{29}H_{34}O_6Na$ 501.2248, found 501.2265 (error −3.4 ppm).

Methyl 2,3,4-tri-*O*-benzyl-6-*O*-[^{13}C]methyl-β-D-glucopyranoside (III*c*)

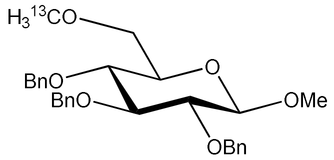

$C_{28}{}^{13}CH_{34}O_6$
479.58 g/mol

To a solution of methyl 2,3,4-tri-*O*-benzyl-β-D-glucopyranoside (0.52 mmol – 1 eq) in dry DMF (5 mL) NaH (1.3 mmol – 2.5 eq) was added at 0 °C under N_2 atmosphere and the mixture was left under stirring for 2 h. [^{13}C]methyl iodide was hence added dropwise (1.04 mmol – 2.0 eq) and stirring continued for 6 h. The reaction was quenched by addition of MeOH, and the mixture was co-evaporated with toluene, diluted with EtOAc and washed with H_2O and brine. The organic fraction was dried over Na_2SO_4 and concentrated under vacuum to afford a yellow oil. The product was purified by flash column chromatography (*Rf*: 0.43, petroleum ether/ethyl acetate 8:2) yielding a colorless oil (79 % yield). ^{1}H NMR (400 MHz, $CDCl_3$): δ 7.37 – 7.23 (*m*, 15H, Ar (Bn)), 4.95 – 4.57 (*m*, 6H, CH_2 (Bn)), 4.30 (*dd*, 1H, $J_{H1,H2}$ = 7.78, H-1), 3.64 (*m*, 1H, H-3), 3.63 (*m*, 1H, H-6a), 3.60 (*m*, 1H, H6b), 3.59 (*m*, 1H, H-4), 3.56 (*s*, 3H, CH_3), 3.44 (*dd*, 1H, $J_{H1,H2}$ = 7.78, H-2), 3.41 (*m*, 1H, H-5), 3.37 (*d*, 3H, $^1J_{CH}$ = 141 Hz, $^{13}CH_3$). ^{13}C NMR (100 MHz, $CDCl_3$): δ 139.0, 138.9, 138.5, 128.8, 128.7, 128.4, 128.3, 128.2, 128.1, 127.92, 127.87 (Ar), 105.1 (C1), 84.9 (C3), 82.6 (C2), 78.0 (C4), 75.9 (CH_2), 75.4 (CH_2), 75.1 (CH_2), 75.0 (*d*, $^3J_{CC}$ = 3.8 Hz, C-5), 71.6 (*d*, $^2J_{CC}$ = 1.9 Hz, C-6), 59.7 (6-*O*-$^{13}CH_3$), 57.4 (CH_3). ESI-HRMS: $[M + Na]^+$ *m/z* calculated for $C_{28}{}^{13}CH_{34}O_6Na$ 502.2281, found 502.2306 (error −4.9 ppm).

Methyl 6-*O*-methyl-β-D-glucopyranoside (IV)

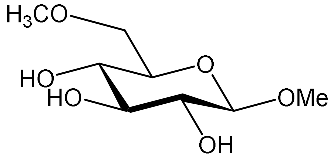

$C_8H_{16}O_6$
208.21 g/mol

To a solution of methyl 2,3,4-tri-*O*-benzyl-6-*O*-methyl-β-D-glucopyranoside (0.21 mmol – 1 eq) and $Pd(OH)_2/C$ (10% w/w) in methanol (3 mL) at r.t. Et_3SiH (0.63 mmol – 3.0 eq) was added portionwise whereafter the progress of the reaction was monitored by TLC. The mixture was hence filtrated through a short pad of Celite and concentrated under vacuum to afford a white amorphous solid. The crude product was purified by reverse phase chromatography with a t-C18 Sep-Pak® cartridge and isolated by gel permeation chromatography using an ÄKTA™ system equipped with a Superdex™ column (GE Healthcare, Uppsala, Sweden) to yield a white powder quantitatively. ^{1}H NMR (600 MHz, D_2O): δ 4.375 (*d*, 1H, $J_{H1,H2}$ = 8.02 Hz, H-1), 3.793 (*dd,* 1H, $J_{H5,H6a}$ = 2.09 Hz, $J_{H6a,H6b}$ = −11.26 Hz, H-6a), 3.644 (*dd*, 1H, $J_{H5,H6b}$ = 6.35 Hz, $J_{H6a,H6b}$ = −11.26 Hz, H-6b), 3.570 (*ddd*, 1H, $J_{H4,H5}$ = 9.86, $J_{H5,H6a}$ = 2.09 Hz, $J_{H5,H6b}$ = 6.35 Hz, H-5), 3.569 (*s*, 3H, CH_3), 3.485 (*dd*, 1H, $J_{H2,H3}$ = 9.42 Hz, $J_{H3,H4}$ = 9.20, H-3), 3.417 (*s*, 3H, 6-*O*-CH_3), 3.379 (*dd*, 1H, $J_{H3,H4}$ = 9.20, $J_{H4,H5}$ = 9.86, H-4), 3.260 (*dd*, 1H, $J_{H1,H2}$ = 8.02 Hz, $J_{H2,H3}$ = 9.42 Hz, H-2). ^{13}C NMR (150 MHz, D_2O): δ 104.1 (C-1), 76.5 (C-3), 75.3 (C-5), 73.8 (C-2), 71.9 (C-6), 70.5 (C-4), 59.4 (6-*O*-CH_3), 58.1 (CH_3). ESI-HRMS: $[M + Na]^+$ *m/z* calculated for $C_8H_{16}O_6Na$ 231.0839, found 231.0845 (error −2.7 ppm).

Methyl 6-*O*-[^{13}C]methyl-β-D-glucopyranoside (IV*c*)

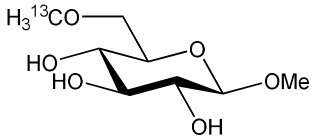

$C_7{}^{13}CH_{16}O_6$
209.20 g/mol

To a solution of methyl 2,3,4-tri-*O*-benzyl-6-*O*-[^{13}C]methyl-β-D-glucopyranoside (0.21 mmol – 1 eq) and $Pd(OH)_2/C$ (10% w/w) in methanol (3 mL) at r.t. Et_3SiH (0.63 mmol – 3.0 eq) was added portionwise whereafter the progress of the reaction was monitored by TLC. The mixture was hence filtrated through a short pad of Celite and concentrated under vacuum to afford a white amorphous solid. The crude product was purified by reverse phase chromatography with t-C18 Sep-Pak® cartridges and isolated by gel permeation chromatography using an ÄKTA™ system equipped with a Superdex™ column (GE Healthcare, Uppsala, Sweden) to yield a white powder quantitatively. ^{1}H NMR (600 MHz, D_2O): δ 4.376 (*d*, 1H, $J_{H1,H2}$ = 8.01 Hz, H-1), 3.793 (*ddd,* 1H, $J_{H5,H6a}$ = 2.24 Hz, $J_{H6a,H6b}$ = −11.28 Hz, $J_{C,H6a}$ = 2.69 Hz, H-6a), 3.645 (*ddd*, 1H, $J_{H5,H6b}$ = 6.56 Hz, $J_{H6a,H6b}$ = −11.28 Hz, $J_{C,H6b}$ = 3.16 Hz, H-6b), 3.572 (*ddd*, 1H, $J_{H4,H5}$ = 9.78, $J_{H5,H6a}$ = 2.24 Hz, $J_{H5,H6b}$ = 6.56 Hz, H-5), 3.570 (*s*, 3H, CH_3), 3.486 (*dd*, 1H, $J_{H2,H3}$ = 9.44 Hz, $J_{H3,H4}$ = 9.22, H-3), 3.415 (*d*, 3H, 6-*O*-$^{13}CH_3$, $^{1}J_{CH}$ = 143 Hz), 3.380 (*dd*, 1H, $J_{H3,H4}$ = 9.22, $J_{H4,H5}$ = 9.78, H-4), 3.261 (*dd*, 1H, $J_{H1,H2}$ = 8.01 Hz, $J_{H2,H3}$ = 9.44 Hz, H-2). ^{13}C NMR (150 MHz, D_2O): δ 104.1 (C-1), 76.4 (C-3), 75.3 (*d*, $^{3}J_{CC}$ = 3.4 Hz, C-5), 73.8 (C-2), 71.9 (*d*, $^{2}J_{CC}$ = 1.5 Hz, C-6), 70.5 (C-4), 59.3 (6-*O*-$^{13}CH_3$), 58.1 (CH_3). ESI-HRMS: $[M + Na]^+$ *m/z* calculated for $C_7{}^{13}CH_{16}O_6Na$ 232.0872, found 232.0865 (error 3.1 ppm).

^{1}H and ^{13}C NMR spectra of compound II

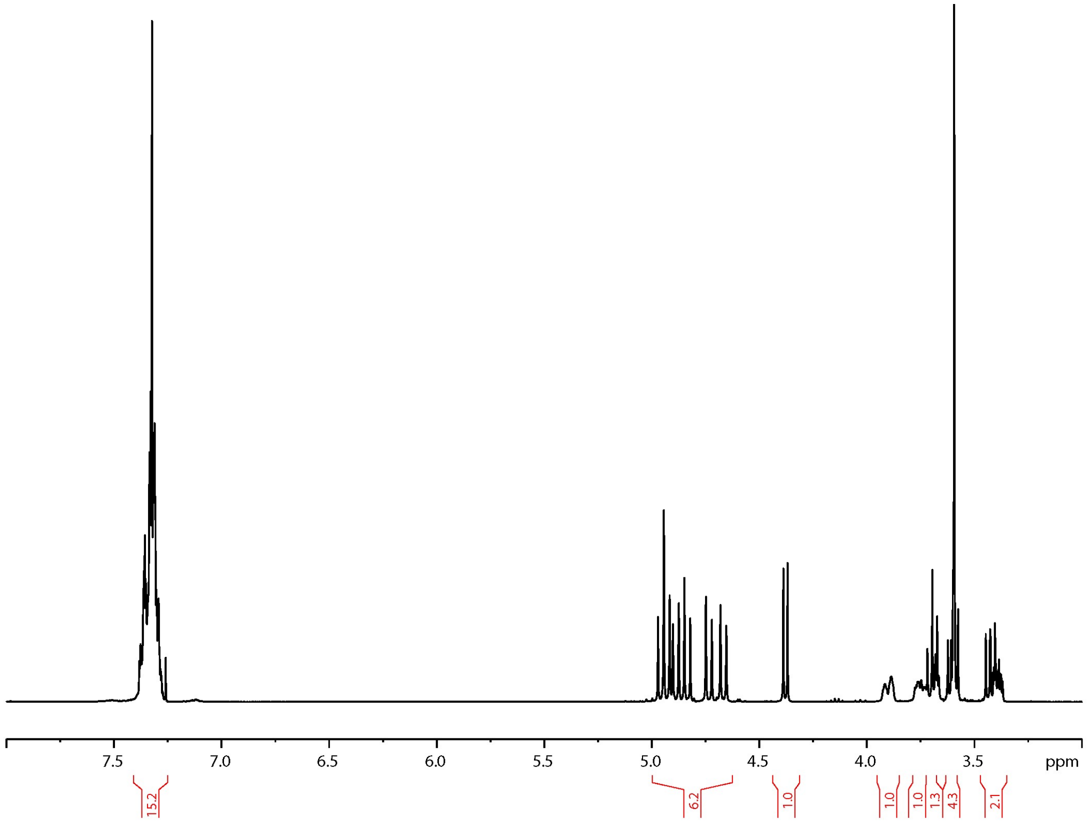

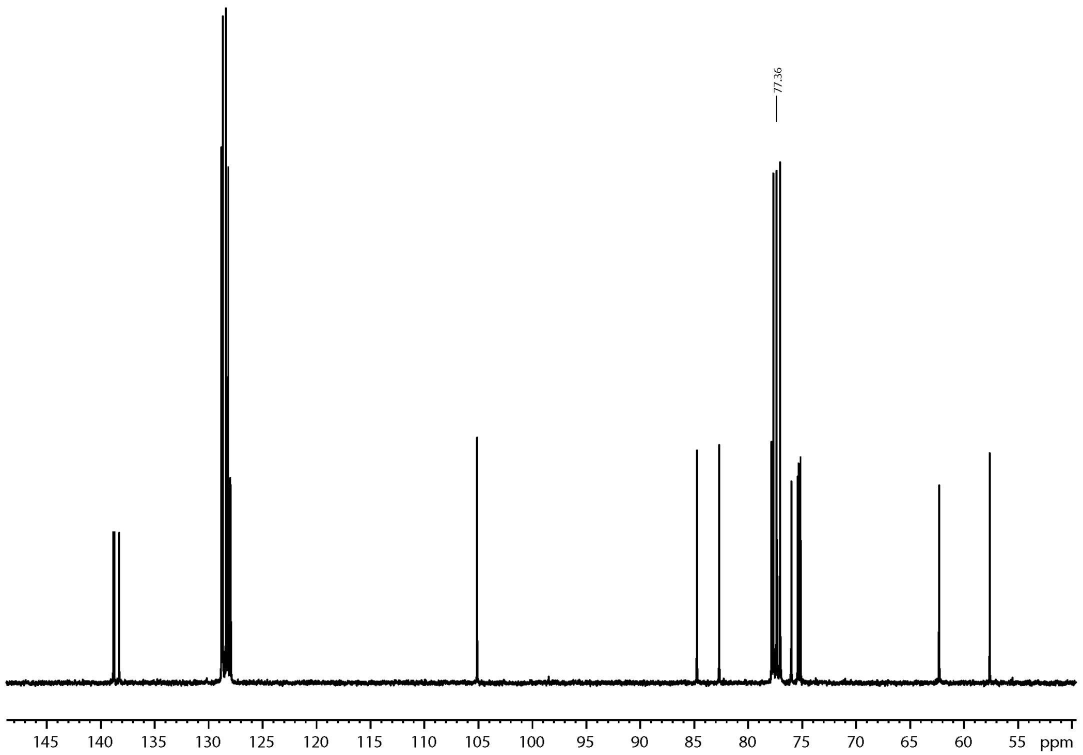

^{1}H and ^{13}C NMR spectra of compound III

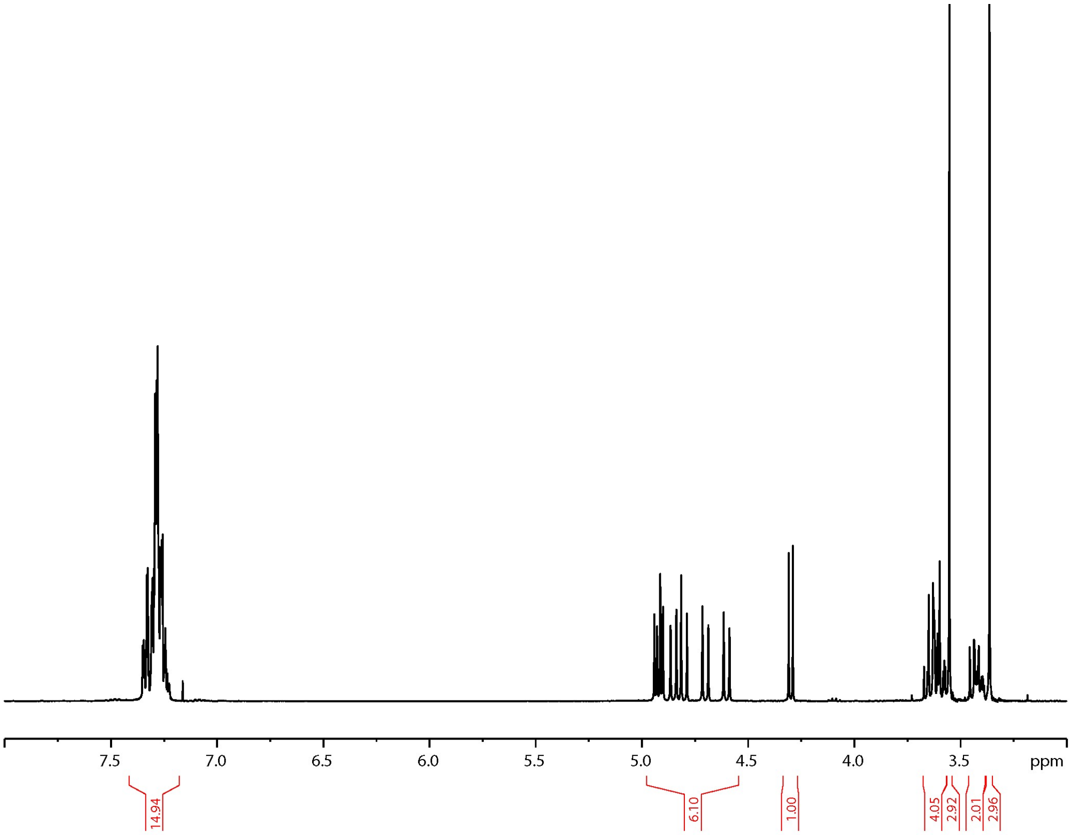

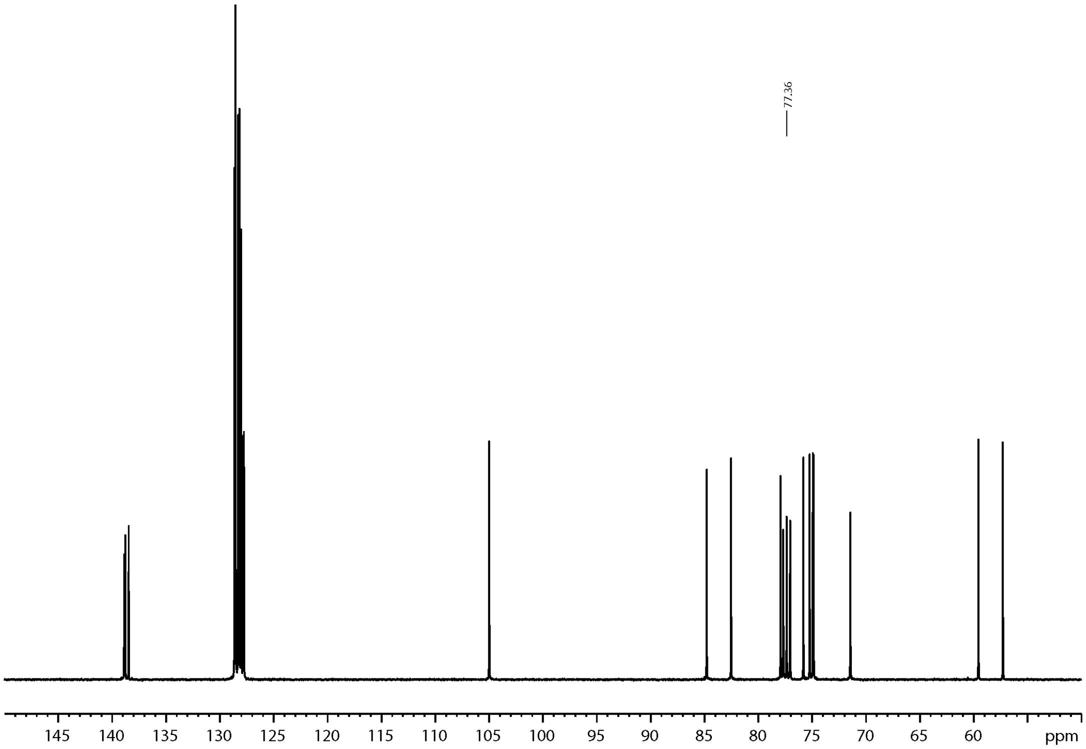

^{1}H and ^{13}C NMR spectra of compound III*c*

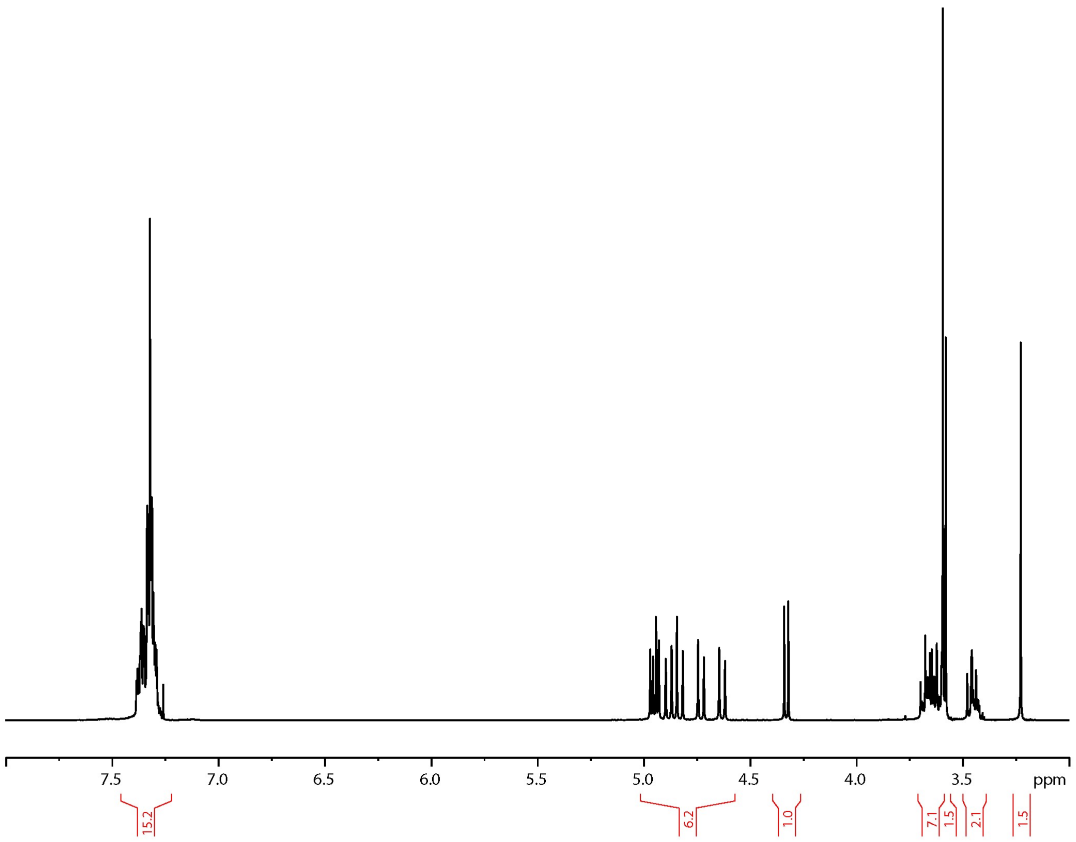

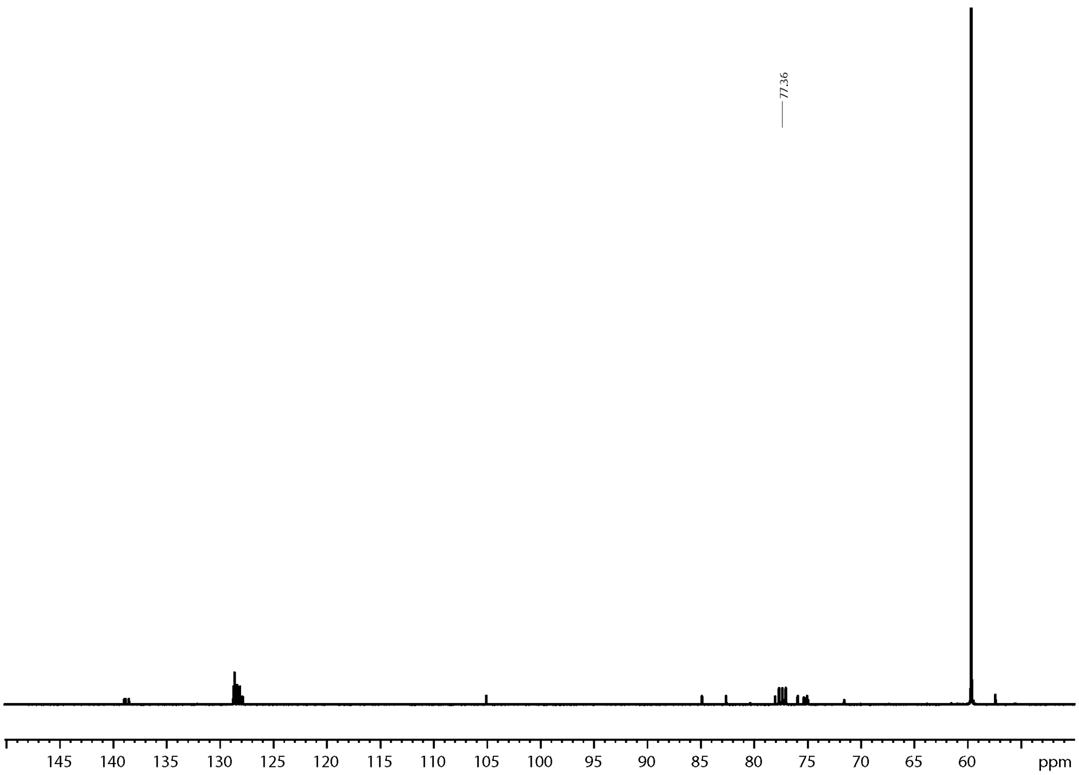

^{1}H and ^{13}C NMR spectra of compound IV

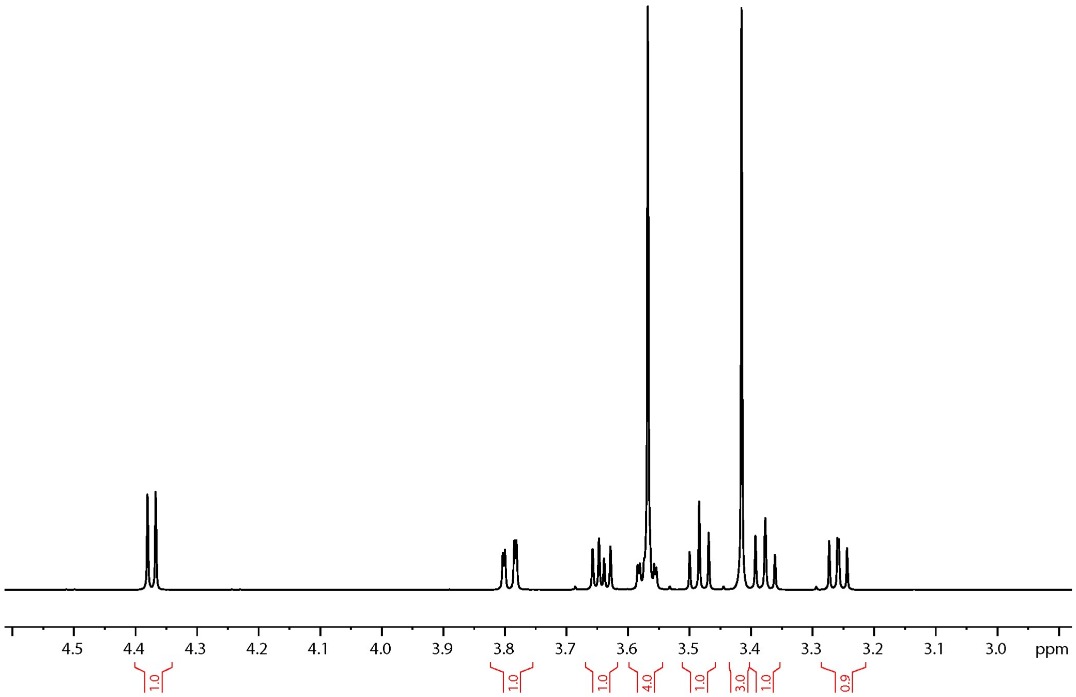

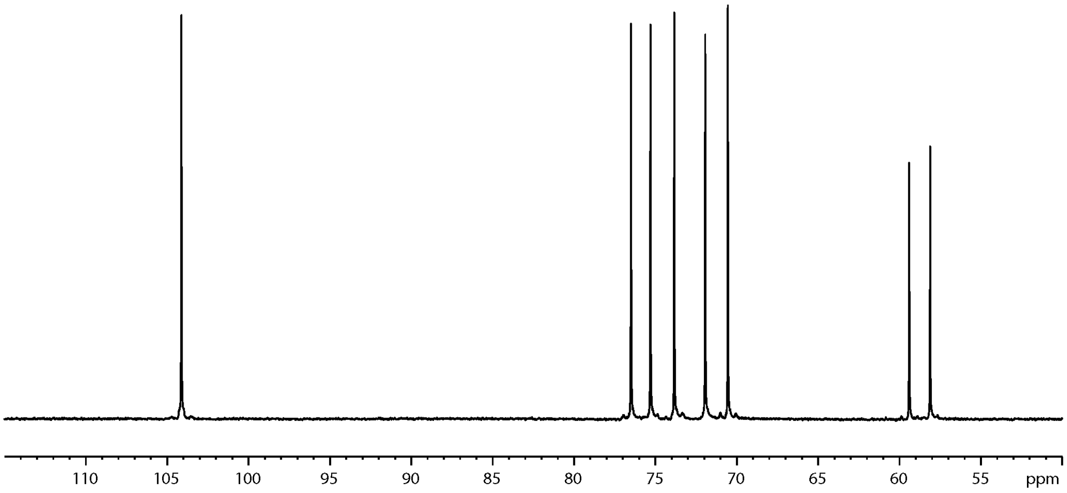

^{1}H and ^{13}C NMR spectra of compound IV*c*

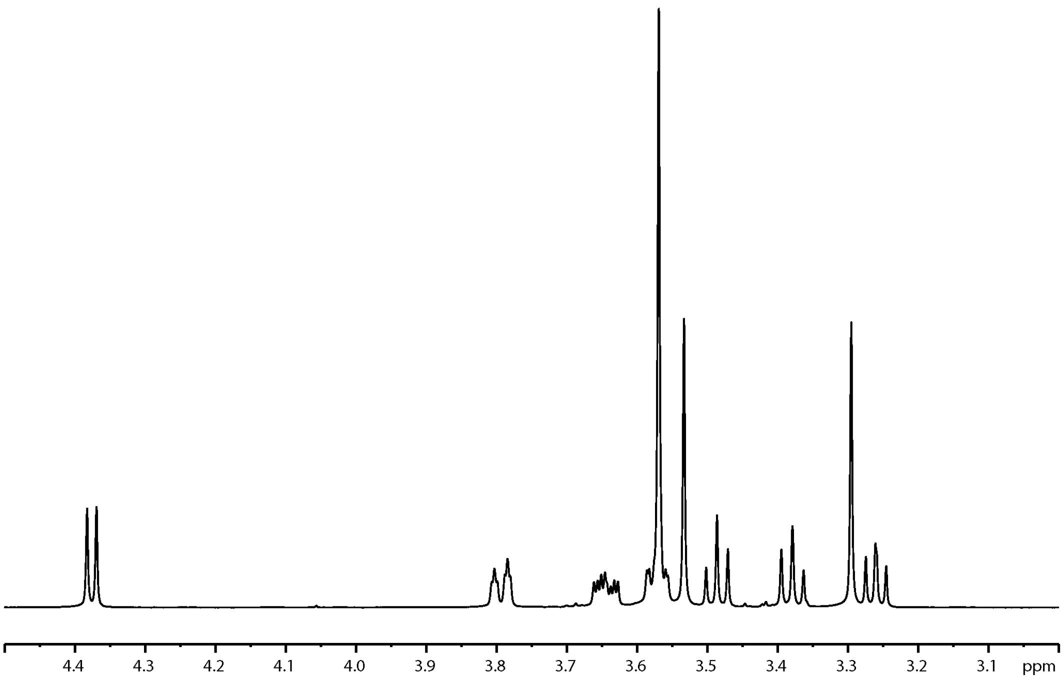

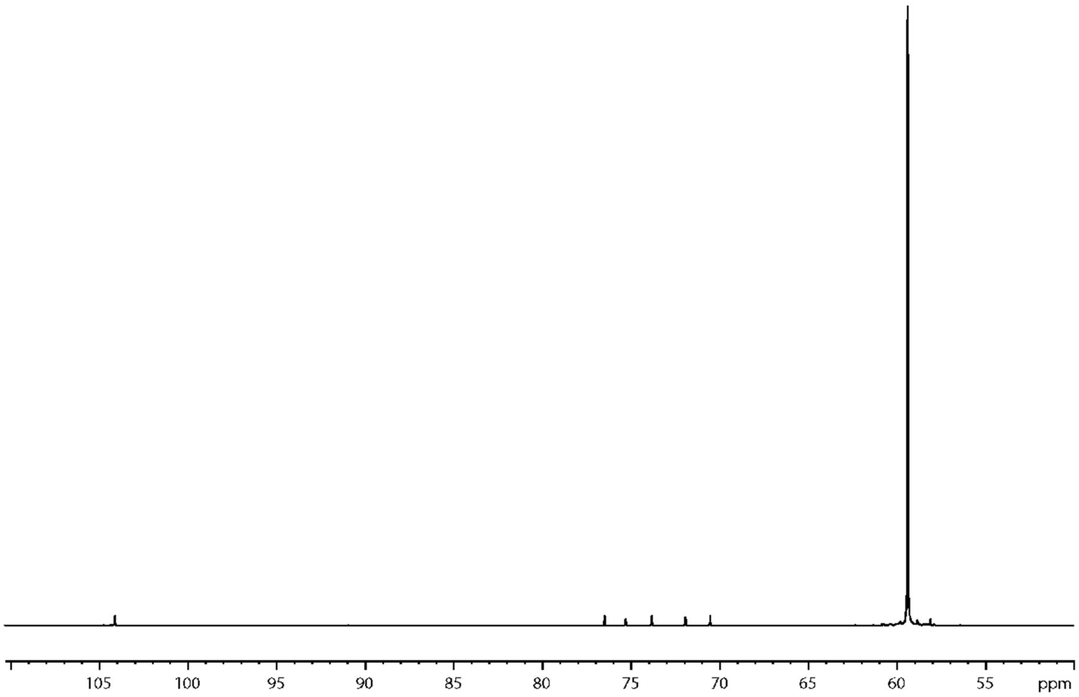

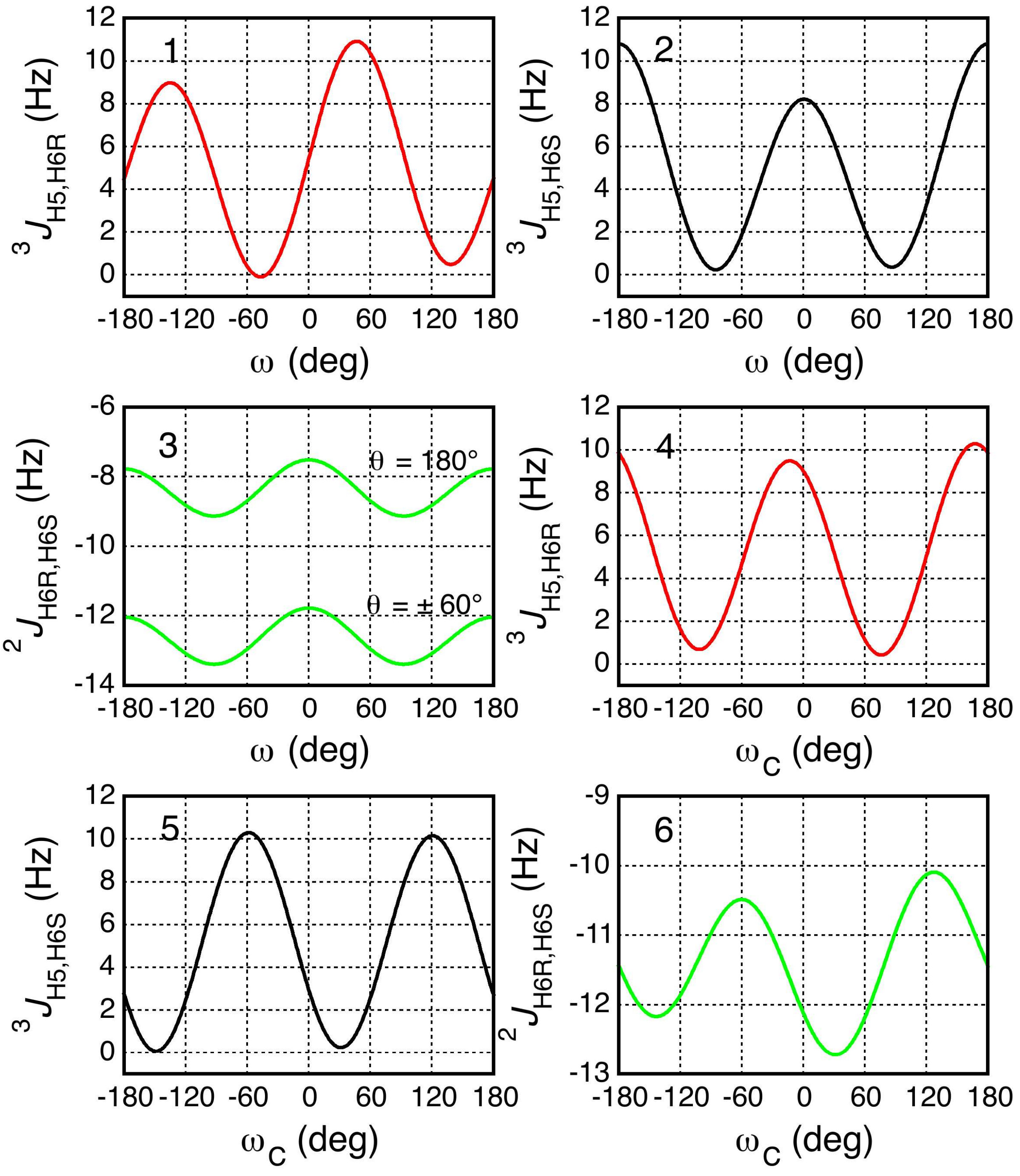

Karplus-type relationships for eqns 1 – 6.

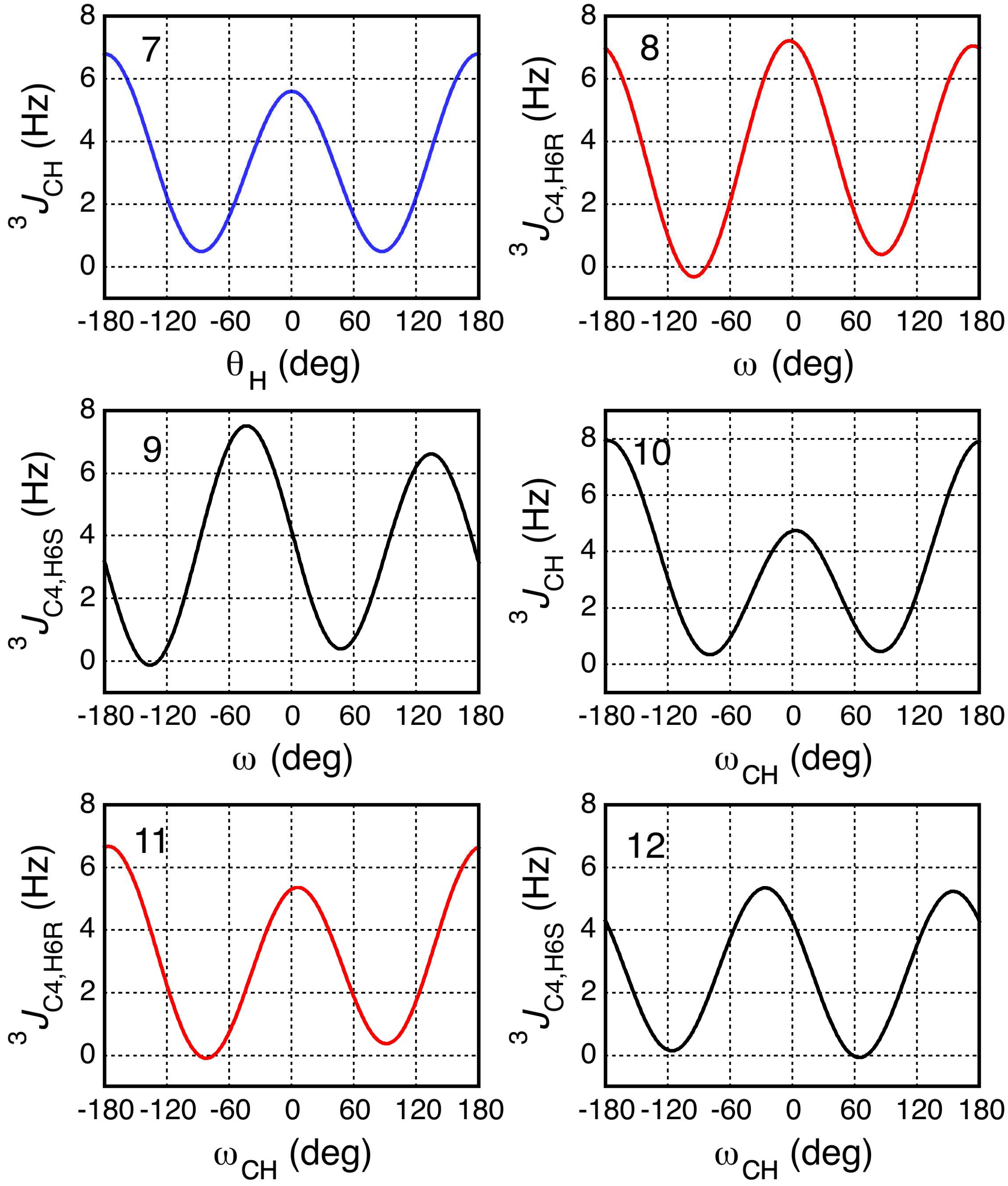

Karplus-type relationships for eqns 7 – 12.
